# Upcycling Quince Peel into Bioactive Ingredients and Fiber Concentrates through Multicomponent Extraction Processes

**DOI:** 10.3390/antiox12020260

**Published:** 2023-01-23

**Authors:** Alexis Pereira, Mikel Añibarro-Ortega, Marina Kostić, António Nogueira, Marina Soković, José Pinela, Lillian Barros

**Affiliations:** 1 Centro de Investigação de Montanha (CIMO), Instituto Politécnico de Bragança, Campus de Santa Apolónia, 5300-253 Bragança, Portugal; 2 Laboratório Associado para a Sustentabilidade e Tecnologia em Regiões de Montanha (SusTEC), Instituto Politécnico de Bragança, Campus de Santa Apolónia, 5300-253 Bragança, Portugal; 3 Institute for Biological Research “Siniša Stanković”—National Institute of Republic of Serbia, University of Belgrade, Bulevar despota Stefana 142, 11000 Belgrade, Serbia

**Keywords:** malic acid, phenolic compounds, dietary fiber, sustainable food ingredients, extraction optimization, antioxidant activity, antimicrobial activity

## Abstract

This study aimed to promote the total upcycling of quince (*Cydonia oblonga* Mill.) peel into bioactive extracts (BEs) and fiber concentrates (FCs). The multicomponent extraction processes were optimized using response surface methodology (RSM) coupled with a 20-run experimental design, where the effects of time (1–120 min), temperature (25–95 °C), and EtOH percentage (0–100%) were combined. In addition to the extraction yields, BEs were analyzed for phenolic compounds, organic acids, and other water-soluble constituents, while FCs were characterized for their color and dietary fiber content. Statistically valid theoretical models were obtained by fitting these dependent variables to a quadratic equation and used to predict optimal extraction conditions. Those obtained for phenolic compounds and malic acid were experimentally validated, yielding 9.3 mg/g and 7.6 g/100 g of these bioactive constituents, respectively, and about 51% (*w/w*) FC. These BEs showed in vitro antioxidant activity and antimicrobial effects against foodborne fungi and bacteria, standing out in some aspects in relation to synthetic food additives, mainly the malic acid-enriched BE. Overall, the developed extraction processes allowed valorizing of quince peel in FCs and BEs that could be used as natural fortifiers or preservatives in the formulation of foods, beverages and dietary supplements.

## 1. Introduction

According to the Food and Agriculture Organization (FAO) of the United Nations, global food production must increase in order to meet the demands of the growing world population. Yet, even under the threat of natural resources depletion and climate change, a considerable percentage of the food produced worldwide is lost or wasted along the food value chain, representing a missed opportunity to improve food security and human nutrition with serious economic, environmental, and social impacts [1]. Despite international efforts to reduce food wastage and promote circular economy and sovereignty, more investments are needed to encourage these sustainability practices and to show that they can generate multi-level benefits [2].

Nowadays, food waste biorefineries have been proposed as innovative technological solutions to the growing challenge of waste management, giving their high potential to reduce the environmental burden and produce bio-based chemicals, materials, and energy [3]. In this way, food waste receives a second chance by entering the value cycle as a renewable biorefinery feedstock to obtain high value-added products. There are studies focused on multi-component cascade biorefinery processes for orange [4], potato [5], and pomegranate [6] peels, among other fruit and vegetable waste. Although a sequential biorefinery process has already been implemented for the valorization of quince (*Cydonia oblonga* Mill.) peel and seeds through pectin extraction and subsequent pyrolysis, a process from which bioproducts or biofuels can be obtained [7], the upcycling of industrial waste from this acidic and astringent fruit through sustainable biorefinery concepts deserves further attention.

Quince is widely used for the production of jams, jellies, marmalade, liqueurs, and other sweet foods. Its edible pulp contains high levels of simple sugars, polysaccharides, and organic acids, while lipids and proteins are found in smaller amounts [8]. In turn, its peel is particularly rich in fructose, fiber, malic acid, and potassium [9]. Regarding phenolic compounds, hydroxycinnamic acid derivatives (mainly, 3-caffeoylquinic and 5-caffeoylquinic acids) and polymeric procyanidins (or flavan-3-ols) have been described in whole pitted quince [10] and its peel [9]. Both quince pulp and peel extracts have antioxidant activity, and peel extracts can be even more effective in scavenging free radicals and inhibiting the growth of some microorganisms than pulp extracts [11,12]. These studies highlighted the bioactive potential of quince peel and its potential to be reintroduced in the value cycle as a source of natural antioxidants, antimicrobials, acidulants, and flavor enhancers for food and beverage formulation, among other applications.

The extraction is a crucial step in the isolation of valuable compounds from quince peel or other food waste, but it must be rethought to achieve the complete valorization of this feedstock. Although most studies on the valorization of agri-food waste use only the filtrate or supernatant from the extraction, the resulting residues should also be used as potential sources of food value ingredients, such as dietary fiber [9,13,14]. Furthermore, since there is no standard extraction procedure due to the wide variety of waste and bioactive molecules it may contain, it is critical to develop targeted extraction processes for particular constituents from specific food waste, thus contributing to efficient utilization and circular bioeconomy. The effects of relevant factors or independent variables on the extraction process can be evaluated using suitable optimization tools, such as response surface methodology (RSM). RSM involves design, modelling, and optimization steps, and allows the development of predictive models that summarize the behavior of the response(s) variable(s) under the tested experimental conditions [15], allowing one to determine the conditions that maximize the extraction yield, minimizing the degradation of target compounds and making it possible to save solvent and energy.

This study was performed to evaluate the upgrading potential of quince peel for simultaneous production of bioactive extracts (BEs) and dietary fiber concentrates (FCs) for future application as natural food preservatives and fortifiers. For this, the plant material was processed under an RSM-couped 20-run central composite rotatable design (CCRD) and the extraction supernatants (named BEs) were analyzed for phenolic compounds and malic acid, among other water-soluble constituents, while the extraction residues (named FCs) were characterized for their dietary fiber content and color parameters. After determining optimal extraction conditions, the model-predicted outcomes were experimentally validated and the resulting phenolic and malic acid-enriched BEs were characterized for their in vitro antioxidant activity and antimicrobial effects.

## 2. Materials and Methods

### 2.1. Plant Material

Quince (*Cydonia oblonga* Mill.) peels were supplied by local farmers from the Bragança region, Portugal, in October 2020, who grow this fruit for homemade marmalade production. The peel sample containing 74.6 ± 0.2% of water was lyophilized to constant weight, ground with a domestic mill until passing through a 20-mesh sieve, and kept vacuum-packaged at −20 °C until use.

### 2.2. Experimental Design for Extraction Optimization

The independent variables *X*_1_ (time, *t*, 1–119 min), *X*_2_ (temperature, *T*, 26–94 °C), and *X*_3_ (EtOH percentage, *S*, 0–100%, *v/v*) were combined in a CCRD composed of 8 factorial points, 6 axial or star points, and 1 center point replicated six times and investigated using Design-Expert software, 11 (Stat-Ease, Inc., Minneapolis, MN, USA). These independent variables and their range of values were selected based on previous studies [16,17]. The CCRD allowed both axial and factorial points to have the same radial distance from the center and, therefore, the same prediction error magnitude. The natural and coded values are presented in Table 1. The 20 experimental runs were randomized to minimize unexpected variability. For each design point, mean values (*n* = 6) were used as observed responses.

### 2.3. Extraction and Preparation of BEs and FCs

**Figure 1:**
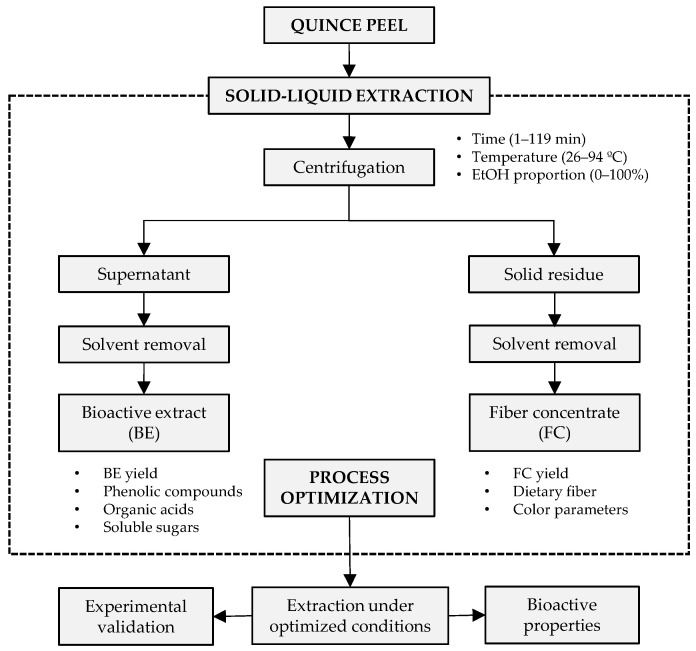
Flowchart of the steps involved in the preparation of bioactive extracts (BE) and fiber concentrates (FC) from quince peel and experimental responses considered for process optimization.

The extraction was performed in a thermostated water bath using sealed vessels to avoid solvent evaporation. Sample weights (1.8 g) were mixed with 30 mL of solvent (0–100% EtOH) and stirred at five levels of time (1–119 min) and temperature (26–94 °C) according to the 20-run design matrix. After extraction, the mixtures were centrifuged at 4000× *g* for 10 min and the supernatants (BEs) and solid residues (FCs) were collected (Figure 1). An aliquot of the supernatants was used to determine the BE yield (%, *w/w*) and the remaining portion was concentrated under reduced pressure to remove EtOH and lyophilized for further analysis of phenolic compounds, organic acids, and soluble sugars. The solid residues were oven-dried at 60 °C until constant weight for subsequent determination of FC yield and dietary fiber content and measurement of color parameters.

### 2.4. Determination of Experimental Responses

#### 2.4.1. Extraction Yields

The BE and FC yields (%, *w/w*) were determined gravimetrically. For BE, 4 mL of each extraction supernatant (obtained in 2.3) was placed into calcined porcelain crucibles and the solvent was removed at 105 °C for at last 24 h until constant weight. The FC yields were determined by weight difference after oven-drying at 60 °C.

#### 2.4.2. Phenolic Compounds

The BEs were redissolved to 6 mg/mL in 20% EtOH, filtered through 0.22-μm syringe filters, and analysis in a Dionex Ultimate 3000 UPLC system (Thermo Scientific, San Jose, CA, USA) with a diode array detector (DAD, using 280 and 370 nm as preferred wavelengths) and a LTQ XL linear ion trap mass spectrometer (MS, Thermo Finnigan, San Jose, CA, USA) equipped with an electrospray ionization (ESI) source [18]. Chromatographic separation was made in a Waters Spherisorb S3 ODS-2 C18 column (4.6 mm × 150 mm, 3 µm; Waters, Milford, MA, USA). Compounds were identified by comparison of their retention times and UV-vis and mass spectra with those of available standards and data from the literature. The concentration (mg/g BE) of the identified compounds was calculated by interpolating the peak areas on 7-level calibration curves (*r*^2^ ≥ 0.999) constructed with standards of chlorogenic acid (*y* = 168,823*x* – 161,172; limit of detection (LOD) = 0.20 µg/mL; limit of quantification (LOQ) = 0.68 µg/mL), *p*-coumaric acid (*y* = 301,950*x* + 6966.7; LOD = 0.68 μg/mL; LOQ = 1.61 μg/mL), catechin (*y* = 84,950*x* – 23,200; LOD = 0.17 μg/mL; LOQ = 0.68 μg/mL), and quercetin-3-*O*-glucoside (*y* = 34,843*x* – 160,173; LOD = 0.21 µg/mL; LOQ = 0.71 µg/mL) purchased from Extrasynthese (Genay Cedex, France). Compounds were thus expressed in equivalents of their basic constituent or similar compound (i.e., standard used in quantification).

#### 2.4.3. Organic Acids

The BEs were redissolved to 6 mg/mL in metaphosphoric acid, filtered through 0.2-μm syringe filters, with analysis in an ultra-fast liquid chromatography (UFLC) system (Shimadzu 20A series, Kyoto, Japan) coupled to a photodiode array (PDA) detector as previously described [19]. Chromatographic separation was achieved in reverse phase on a C18 column (250 mm × 4.6 mm, 5 µm; Phenomenex, Torrance, CA, USA). Detection was done on PDA at 215 and 245 nm. The detected compounds were identified by chromatographic comparisons with standards and quantified (g/100 g BE) by interpolating the peak areas in calibration curves (*r*^2^ ≥ 0.994) constructed with oxalic acid (*y* = 9 × 10^6^*x* + 377.946; LOD = 12.55 µg/mL; LOQ = 41.82 µg/mL), quinic acid (*y* = 612.327*x* + 16.563; LOD = 24.18 µg/mL; LOQ = 80.61 µg/mL) and malic acid (*y* = 912.441*x* + 92.665; LOD = 35.76 µg/mL; LOQ = 119.18 µg/mL) standards acquired from Sigma-Aldrich (Saint Louis, MO, USA).

#### 2.4.4. Soluble Sugars

The BEs were redissolved to 6 mg/mL in distilled water, filtered through 0.2-µm syringe filters, and analyzed in by HPLC with refraction index detection as previously described [20]. Chromatographic separation was achieved with a Eurospher 100-5 NH_2_ column (4.6 × 250 mm, 5 mm, Knauer, Berlin, Germany), using acetonitrile/water 70:30 (*v/v*) as mobile phase. Soluble sugars were identified by chromatographic comparisons with standards and quantified (g/100 g BE) by interpolating the peak areas in calibration curves (*r*^2^ ≥ 0.999) constructed with fructose (*y* = 1.04*x*; LOD = 0.05 mg/mL; LOQ = 0.18 mg/mL), glucose (*y* = 0.935*x*; LOD = 0.08 mg/mL; LOQ = 0.25 mg/mL), and sucrose (*y* = 0.977*x*; LOD = 0.06 mg/mL; LOQ = 0.21 mg/mL) standards acquired from Sigma-Aldrich (St. Louis, MO, USA).

#### 2.4.5. Dietary Fiber

The dietary fiber content of the FCs was determined by an enzymatic-gravimetric method (AOAC 985.29) [21]. Briefly, the FCs (250 mg) were gelatinized with heat-stable α-amylase (pH 6.0, 95 °C water bath for 15 min) and then enzymatically digested with protease (pH 7.5, 60 °C water bath for 30 min) and amyloglucosidase (pH 4.5, 60 °C water bath for 30 min) to remove protein and starch (kit from Sigma-Aldrich, Saint Louis, MO, USA). After overnight EtOH precipitation followed by filtration, the residues were successively washed with 78% EtOH, 95% EtOH, and acetone, and then oven-dried at 105 °C and weighed. The protein (AOAC 920.152) and ash (AOAC 940.26) contents were determined by macro-Kjeldahl nitrogen analysis (N × 6.25) or incineration at 525 °C, respectively, for subsequent calculation of the dietary fiber content (g/100 g FC).

#### 2.4.6. Color Parameters

The FC color was measured with a colorimeter (model CR-400; Konica Minolta Sensing Inc., Japan) calibrated with a standard white tile. The parameters L* (lightness, from ^(0)^ black to ^(100)^ white), a* (chromaticity from ^(–)^ green to ^(+)^ red), and b* (chromaticity from ^(–)^ blue to ^(+)^ yellow) were measured as previously described by the authors [22]. For visual representation, the CIELAB color values were converted to RGB (red, green, blue) color.

### 2.5. Extraction Process Modelling and Statistical Verification of the Models

The response variables considered for the optimization of the extraction process were grouped into three main groups: (i) bioactive extract (BE) and its main compounds (BE yield (%, *w/w*), Σ phenolic compounds (mg/g BE), Σ phenolic acids (mg/g BE), Σ flavan-3-ols (mg/g BE), and malic acid (g/100 g BE)); (ii)) other water-soluble BE constituents (quinic acid (g/100 g BE), Σ organic acids (g/100 g BE), fructose (g/100 g PE), glucose (g/100 g BE), and Σ soluble sugars (g/100 g BE)); and iii) fiber concentrate (FC) and its properties (FC yield (%, *w/w*), dietary fiber (g/100 g FC), lightness (L*), redness (a*), and yellowness (b*)).

The response surface models were fitted using the quadratic Equation (1):

(1) $$Y=b_{0}+b_{1}X_{1}+b_{2}X_{2}+b_{3}X_{3}+b_{11}X_{1}^{2}+b_{22}X_{2}^{2}+b_{33}X_{3}^{2}+b_{12}X_{1}X_{2}+b_{13}X_{1}X_{3}+b_{23}X_{2}X_{3}$$

where *Y* corresponds to the dependent variable, *X* define the independent variables, *b*_0_ is the constant coefficient, *b*_1_, *b*_2_, and *b*_3_ are linear term coefficients, *b*_11_, *b*_22_, and *b*_33_ are quadratic term coefficients, and *b*_12_, *b*_13_, and *b*_23_ are interaction term coefficients. Subscripts 1, 2, and 3 in each term stand for time (*t*), temperature (*T*), and solvent (*S*), respectively.

Fitting procedures, coefficient estimates, and statistical analysis were performed using Design-Expert software. The significance of the models and their terms was assessed by an analysis of variance (ANOVA) at a 95% confidence level, and only the significant terms or those necessary for the hierarchy were considered in the fitting procedures. The lack-of-fit test, the coefficients *R*^2^ and *R*²_adj_ and the adequate precision were used to assess the model-fitting adequacy. Design-Expert was also used to generate the 2D and 3D plots.

### 2.6. Experimental Validation of the Models and Evaluation of Bioactive Properties of BEs Obtained under Optimized Conditions

Enriched quince peel extracts were obtained under the optimal settings obtained for phenolic compounds, malic acid, and both phytoconstituents. The extraction and analysis were caried out as described in the previous sections and the quantitative results were used for experimental validation of the theoretical models, by comparing experimental and model-predicted data. The BE and FC yields and the FC color parameters were also evaluated, as well as the BE antioxidant and antimicrobial activities. These experiments were performed in triplicate and each sample was measured three times (*n* = 9).

#### 2.6.1. Antioxidant Activity

The ability of the BEs to inhibit oxidative hemolysis and the formation of thiobarbituric acid reactive substances (TBARS) such as the aldehyde malondialdehyde (MDA) was evaluated in vitro as described below. Trolox and the food additives calcium ascorbate (E302) and sodium metabisulfite (E223) were tested as positive controls.

For TBARS, a porcine brain tissue solution (1:2, *w/v*; 0.1 mL) was mixed with 0.1 mL of FeSO_4_ (10 µM) and 0.1 mL of ascorbic acid (0.1 mM) and incubated with 0.2 mL of BE (0.016–8 mg/mL), E302 (0.01–2.56 mg/mL), E223 (0.01–2.56 mg/mL), or trolox (3.125–100 µg/mL) for 60 min at 37 °C. After adding 0.5 mL of trichloroacetic acid (28% *w/v*) and 0.38 mL of thiobarbituric acid (2%, *w/v*), the mixtures were incubated at 80 °C for 20 min and the color intensity provided by MDA-TBA adducts was monitored (Specord 200 spectrophotometer, Analytik-Jena, Jena, Germany) at 532 nm. Half-maximal effective concentration (EC_50_) values (µg/mL) were calculated as previously described by the authors [23].

For oxidative hemolysis inhibition, a red blood cell (RBC) solution (2.8%, *v/v*; 0.2 mL) prepared in PBS (pH 7.4) was mixed with 0.4 mL of either BE (0.125–4 mg/mL), E302 (0.01–2.56 mg/mL), E223 (0.01–2.56 mg/mL), trolox (7.81–250 µg/mL), PBS (control), or water (baseline) in a 48 well plate. After 10 min pre-incubation at 37 °C with shaking, 0.2 mL of the free radical generator 2,2′-azobis(2-methylpropionamidine) dihydrochloride (AAPH, 160 mM) was added, and the optical density was monitored at 690 nm (Elx800 plate reader, BioTek) over time. Half-maximal inhibitory concentration (IC_50_) values (µg/mL) were calculated for Δ*t* of 60 and 120 min, as previously described by the authors [23].

#### 2.6.2. Antimicrobial Activity

The BEs redissolved in 30% EtOH at 20 mg/mL were screened against the Gram-positive bacteria *Staphylococcus aureus* (ATCC 11632), *Bacillus cereus* (food isolate), and *Listeria monocytogenes* (NCTC 7973) and the Gram-negative bacteria *Escherichia coli* (ATCC 25922), *Salmonella enterica* subsp. *enterica* serovar Typhimurium (ATCC 13311), and *Enterobacter cloacae* (clinical isolate). The micromycetes *Aspergillus fumigatus* (human isolate), *Aspergillus niger* (ATCC 6275), *Aspergillus versicolor* (ATCC 11730), *Penicillium funiculosum* (ATCC 36839), *Penicillium verrucosum* var. *cyclopium* (food isolate), and *Trichoderma viride* (IAM 5061) were used to assess antifungal activity. The microorganisms were obtained from the Mycological laboratory, Department of Plant Physiology, Institute for Biological Research “Siniša Stanković”, National Institute of the Republic of Serbia, University of Belgrade, Serbia.

The lowest extract concentration (mg/mL) that inhibited the visible microbial growth at the binocular microscope (minimum inhibitory concentration, MIC) and the lowest concentration (mg/mL) required to kill the original inoculum (minimal bactericidal or fungicidal concentrations, MBC and MFC, respectively) were determined by the serial microdilution method and the *p*-iodonitrotetrazolium violet (INT) colorimetric assay [24,25]. The antibiotics streptomycin and ampicillin, the antifungals ketoconazole and bifonazole, and the food additives sodium benzoate (E211) and potassium metabisulfite (E224) were used as positive controls, while 30% EtOH was the negative control.

#### 2.6.3. Evaluation of Statistical Differences between Extracts’ Bioactivity

The antioxidant activity results were expressed as the mean ± standard deviation and differences among samples were assessed using one-way analysis of variance (ANOVA). Data normality and variance homogeneity were evaluated by the Shapiro–Wilk and Levene’s tests, respectively. Results were compared using the Tukey’s HSD test. Additionally, a Pearson’s correlation was performed to assess possible correlations between antioxidant activity and BE constituents. Statistical tests were performed at a 5% significance level using SPSS^®^ Statistics, Version 28.0 (IBM Crop, Armonk, NY, USA).

## 3. Results & Discussion

### 3.1. Extraction Yields and Chemical Composition

Previous studies described quince peel as a source of high value-added compounds and aroused interest in upcycling this renewable resource into food-grade ingredients [8,9]. Therefore, after running the experimental design, the most promising constituents identified in the BEs and FCs were used as response variables for extraction process optimization.

#### 3.1.1. Bioactive Extract (BE) Yields

As shown in Table 2, the extraction yield (weight of solids per weight of feed material) ranged from 34.47 to 67.09% (*w/w*) with the runs 14 and 11 of the design, which involved the highest level (α = +1.68) of solvent and the lowest (α = −1.68) of temperature, respectively, combined with medium levels (α = 0) of the other two independent variables. The replicated center point yielded 51 ± 1% (*w/w*). This range of values comprises the extraction yields obtained by Othman et al. [9] for quince peel extracts after a 2 h dynamic maceration with 80% EtOH (53 ± 2%, *w/w*) and a 5 min boiling water extraction (54 ± 1%, *w/w*).

#### 3.1.2. Phenolic Compounds

Phenolic compounds represent the largest group of antioxidants in the human diet. Epidemiological studies and meta-analyses have shown that diets rich in polyphenols offer protection against the development of inflammatory and neurodegenerative diseases and various types of cancer [26,27]. A representative chromatogram of the phenolic profile of quince peel BEs is shown in Figure S1 and the detected compounds are listed in Table S1, together with the acquired chromatographic data. Sixteen compounds were tentatively identified, including five phenolic acids (caffeoylquinic acids), nine flavan-3-ols ((+)-catechin, β-type (epi)catechin dimers, trimers, and tetramers, and a procyanidin with A-type linkage), and two flavonol glycosides (quercetin-*O*-deoxyhexosil-hexoside and kaempferol-*O*-deoxyhexosil-hexoside), which agreed with the profile described by Othman et al. [9]. Other authors also identified these compounds in this species [28,29,30,31].

As shown in Table 2, the total content of phenolic compounds ranged from 6.5 to 10.13 mg/g BE with the 14th and 4th runs of the design, respectively, which also gave rise to the highest and lowest concentrations of phenolic acids and flavan-3-ols. These two classes of compounds accounted for about 34.6 and 40.3% of the phenolic content quantified in the replicated center point of the design, respectively, while flavonol glycosides represented 25.1% of the phenolic fraction. For these phytoconstituents, the 12th run (axial point of highest temperature) and the 9th run (axial point of shortest time) gave rise to the lowest (1.82 mg/g BE) and highest (2.25 mg/g BE) concentrations, respectively, differently from what was observed for the other two phenolic classes. However, not all the identified phenolic compounds described in Table S1 were considered for the calculation of the total contents of the compound groups used in the optimization, as some of them (namely, compounds **7**, **10**, **12**, **14**, and **16**) were found in low concentration. The contents of the quantified phenolic compounds are shown in Table S2.

In general, higher BE yields appeared to be associated with greater recovery of phenolic compounds, organic acids, and soluble sugars (Table 2 and Table S2), as well as the use of medium–low EtOH percentages given the water solubility of these constituents.

**Figure 2:**
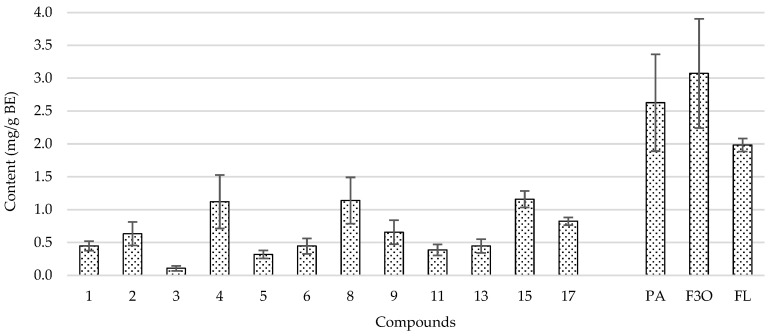
Mean composition in phenolic compounds of quince peel bioactive extracts (BEs) obtained with the six replicates of the center point of the design. **1**: *cis*-3-*O*-caffeoylquinic acid; **2**: *trans*-3-*O*-caffeoylquinic acid; **3**: 3-*O*-*p*-coumaroylquinic acid; **4**: *cis*-5-*O*-caffeoylquinic acid; **5**: *trans*-5-*O*-caffeoylquinic acid; **6**: (+)-catechin; **8**: β-type (epi)catechin trimer; **9**: β-type (epi)catechin tetramer; **11**: β-type (epi)catechin tetramer; **13**: β-type (epi)catechin trimer; **15**: quercetin-*O*-deoxyhexosil-hexoside; **17**: kaempferol-*O*-deoxyhexosil-hexoside. PA: phenolic acids; F3O: flavan-3-ols; FL: flavonols.

As shown in Figure 2, compounds **4** (*cis*-5-*O*-caffeoylquinic acid), **8** (β-type (epi)catechin trimer), and **15** (quercetin-*O*-deoxyhexosil-hexoside) were those detected at higher concentrations (>1.12 mg/g BE) in the quince peel BEs obtained with the replicated central point of the design. Compounds **2** (*trans*-3-*O*-caffeoylquinic acid), **9** (β-type (epi)catechin tetramer), and **17** (kaempferol-*O*-deoxyhexosil-hexoside) ranked second with levels ranging from 0.63 to 0.83 mg/g BE. The remaining compounds were in contractions below 0.5 mg/g BE. The relative proportion of flavan-3-ols, phenolic acids, and flavonols shown in Figure 2 is comparable to that earlier described for quince peel extracts obtained by hydroethanolic maceration and boiling water extraction [9]. However, the total levels detected in the central point BEs were higher than those obtained by Othman et al. [9].

Other studies also described flavan-3-ols (or polymeric procyanidins) as the predominant class of polyphenols in quince, followed by hydroxycinnamic acids and then flavonols [28,29,30]. According to these reports, the content of polymeric procyanidins can be correlated to the quince astringency and bitterness. In turn, caffeoylquinic acids are relevant in the manufacture of quince-based food products, since these phenolic acids are substrates of the catecholase activity of polyphenol oxidase and, therefore, can influence oxidation and color change processes. In turn, 5-*O*-caffeoylquinic acid was previously described as the most prominent hydroxycinnamic acid in quince, mainly in the peel rather than in the pulp, which also stood out for its higher levels of total phenolics and flavonoids [12].

#### 3.1.3. Organic Acids

Organic acids together with sugars are the main soluble components of ripe fruits and have major effects on their palatability and, therefore, on their acceptance by the consumers. Oxalic, quinic, and malic acids were detected in the quince peel extracts. Among these, malic acid predominated in all samples, with levels reaching 6.97 g/100 g BE in the 12th run (Table 2). This dicarboxylic acid has been wildly used in the food, pharmaceutical, and agricultural sectors and as a precursor of industrially important chemicals [32]. Currently, it can be produced by chemical synthesis, enzymatic conversion, biological fermentation, and biologically, by microbial strains. However, its recovery from malic acid-rich agri-food by-products such as quince peel could be an option, but more efficient, environmentally friendly, and economical techniques need to be available at the industrial level.

As shown in Table 2 and Table S2, malic acid represented more than 95% of the total content of organic acids detected in the replicated center point, while the contribution of the other two was minor. Quinic acid ranged from 0.07 to 0.62 g/100 g BE and it was not identified in six extracts (Table S3). In turn, oxalic acid was only detected in half of the runs, with levels ranging from 0.01 to 0.73 g/100 g BE. The higher content was obtained with the 15th run, which consisted of a replica of the center point. Therefore, given the scarcity and inconsistency of experimental data, oxalic acid was not considered as a response variable for the optimization, nor for the calculation of the total organic acid contents.

The results of this work are in line with those of Othman et al. [9] and Rodríguez-Guisado et al. [33], who describe malic acid as the main organic acid in quince peel and pulp juice, respectively. Rodríguez-Guisado et al. [33] described tartaric acid as the second most abundant organic acid, but they did not report quinic acid in their samples. The prevalence of malic and quinic acids in quince peel and pulp was also observed by Silva et al. [34], who also identified citric, ascorbic, shikimic, and fumaric acids. Additionally, the quince pulp tended to contain more organic acids than the peel. These findings highlight the undervalued potential of this plant-derived waste.

#### 3.1.4. Soluble Sugars

Soluble sugars are abundant constituents in plant extracts. However, most studies on the optimization of extraction methods have been focused on one or a restricted number of bioactive compounds, while the sugar composition is often overlooked. As shown in Table S3, the quince peel BEs contained fructose, glucose, and sucrose. Fructose was by far the most abundant carbohydrate (60–79%), followed by glucose (11–25%). These reducing sugars together with sucrose accounted for 44 to 62.8% of the BEs and the lowest levels were reached with the 8th and 14th runs. This sugar profile is in agreement with that firstly described by Othman et al. [9] for quince peel. On the other hand, the quince pulp is well characterized for its composition in sugars and other carbohydrates. Szychowski et al. [35] reported fructose as the major soluble sugar in the pulp of quince samples from Spain, followed by glucose, sorbitol, and traces of sucrose and maltose. Fructose and glucose, and lower amounts of sucrose and maltose, were also quantified in quince juice by Rodríguez-Guisado et al. [33]. Despite the quantitative differences that have been reported, the sugars that prevail in the quince pulp also seemed to be present in the peel.

Since the organoleptic properties of food additives and ingredients can impact the acceptability of foodstuff to which they are added, the sugar/acid ratio must be considered when manufacturing these products, especially from plant sources. The total sugars/total organic acids ratio of the different BEs was quite variable, ranging from 7 (with the 8th run) to 96 (with the 5th run). However, this issue is usually overcome by the dilution effect caused by the low doses commonly used in foodstuffs. Still, it is relevant to consider the excessive consumption of sugar, often hidden in foods under different names, which can result in negative consequences for human health. Therefore, the development of new food ingredients must take this issue into account. For example, the European Regulations specify that the natural colorant beetroot red (E162) must contain at last 0.4% of betanins; the remaining 99.6% is accounted for by sugars, salts, and proteins naturally occurring in red beets but not specified in the regulations. However, the European Food Safety Authority (EFSA) Panel on Food Additives and Nutrient Sources added to Food considers that the E162 could be refined to remove most of these additional constituents [36].

#### 3.1.5. Fiber Concentrates (FCs)

Today, the agri-food sector generates million tons of plant waste rich in dietary fiber, a nutrient that can be recovered and upcycled as a food ingredient [37]. This could be a strategy to achieve circularity and resource efficiency in agri-food systems and to promote adequate intakes of dietary fiber, since a considerable part of the world population does not get the recommended daily amount (25 g/day) [38]. Dietary fiber is a “nutrient of public health concern” that plays an important role in protecting against cardiovascular disease, type 2 diabetes, and cancer, and in improving gastrointestinal health and body weight control [39,40]. In this sense, the quince peel extraction residues or FCs were valorized as a source of dietary fiber.

After extraction, FCs were recovered and the yields (weight of FC per weight of feed material) were determined, as well as the dietary fiber contents and color parameters. These FCs could be used in food fortification, but their color should not significantly impact the appearance of the final food formulations in which they are incorporated [41]. As shown in Table 2, the FC yield ranged from 39.82 to 65.52% (*w/w*) with the 11th and 14th runs, respectively, and higher FC yields tended to be associated with lower percentages of dietary fiber (which constituted 52.46 to 65.55% of FCs). These lower proportions of fiber could be explained by the higher concentrations of soluble sugars and other water-soluble constituents such as pectin. Thus, the FC color may have varied depending on their carbohydrate content, i.e., the higher the sugar percentage, the lighter (higher L*) the FCs. Contrariwise, FCs containing more dietary fiber were darker, like those obtained with the 3rd, 4th, and 12th runs (Table 2), which also resulted from processing at 80 to 95 °C with 20 or 50% EtOH, conditions that may have caused some kind of browning reactions. Indeed, dark colored end-products can result from Millard-type reactions between malic acid and fructose at moderate temperatures [42]. While the L* values (34.09 to 70.62, from darker to lighter) were the ones that varied the most, those of a* (6.31 to 8.51, redness) and b* (11.59 to 22.31, yellowness) did not change so much for the 20 FCs.

In a previous work, Afifi et al. optimized the extraction of fiber from date (*Phoenix dactylifera* L.) seeds following a similar methodological approach [43]. The authors obtained FC yields ranging from 25.40 to 74.13% and fiber contents from 22.35 to 79.37 g/100 g FC when processing the samples at 45 to 65 °C for 60 to 180 min with 25 to 75% EtOH. Interestingly, higher FC yields were associated with lower dietary fiber contents and vice versa, which agrees with the results of the present study. In general, the FCs obtained from date seeds were darker (L* values lower than 48) and reddish (a* values up to 15.14) than those from quince peel (Table 2).

### 3.2. Models Fitting and Statistical Verification

RMS is suitable for optimizing extraction processes involving one or more response variables and allows one to determine optimal processing conditions with a reduced number of experimental trials, when compared with one-factor-at-a-time approaches which do not take the interaction between independent variables into account [44]. When developing polynomial models capable of predicting and understanding the effects of process-independent variables on a given response, it is necessary to assess the accuracy of their fitting to the experimental data. In this work, the response values in Table 2 and Table S2 were fitted to Equation (1) using Design-Expert software, but only the significant parameters (*p* < 0.05) or those necessary for the hierarchy were used.

The model coefficients obtained for the different response variables are shown in Table 3 and Table S3, as also are the statistical data of the modeling. The presented parametric values translate the linear, quadratic, and interaction effects of time, temperature, and solvent (EtOH percentage) and denote the expected change in response per unit change in factor value when all remaining independent variables are held constant. The higher the coefficient value (regardless of the sign), the more marked is the variable effect. Therefore, the complexity of the extraction trends can be illustrated by the developed models. The intercept corresponds to the expected mean value of the response variable when all factors are equal to zero (*X* = 0), i.e., the predicted average response at the center point.

As shown in Table 3, the equation models developed for BE yield, phenolic classes, and malic acid presented significant F-values (*p* < 0.001) and a non-significant lack-of-fit (*p* > 0.05), which indicated that these models adequately describe the effects of the independent variables on the target responses. In all cases, the coefficients *R*^2^ and Adj *R*^2^ were higher than 0.924 and 0.889, respectively, indicating that the variability of each response is explained by the independent variables involved in the extraction process. In addition, adequate precision, which is a measure of the signal-to-noise ratio, had values greater than 17 (values > 4 indicate adequate model discrimination). The high degree of accuracy and reliability was also demonstrated by the low values of the coefficient of variation, which is a measure of the residual variation of the data relative to the size of the mean. Thus, these models were statistically reliable for navigating the design space and subsequent optimization of the extraction of valuable constituents from quince peel.

Significant (*p* ≤ 0.006) model equations were also developed for the other BE constituents, namely, quinic acid and soluble sugars (Table S4). Among these models, those constructed for citric acid and sucrose showed a higher coefficient of variation and a lower model F-value, respectively. However, better statistical results were obtained for the models obtained for total organic acids and total soluble sugars. Thus, the levels of these constituents in the BEs can be predicted when maximizing the extraction of target bioactive compounds.

Table 3 also shows the statistical data of the models developed for FC yield, dietary fiber, and some color parameters. The model developed for a* (redness) presented an F-value of 4.63, while those of the other models were greater than 28. Its adequate precision and coefficient of variation were not so good either. Still, all models were significant (*p* < 0.05) and the lack-of-fit was non-significant (*p* > 0.05).

### 3.3. Effect of the Independent Variables and Optimal Extraction Conditions

The effects of time (*t*), temperature (*T*), and solvent (*S*) on the different response variables, translated by the model coefficients in Table 3 and Table S3, are discussed below. To visually illustrate the extraction trends, 3D and 2D plots were generated, in which the unplotted factor or factors were kept constant at their individual optimum value. For numerical optimization, to determine the conditions that meet the desired objective (yield maximization), the three independent variables were set within the experimental domain and each target response was “maximized”, keeping the standard error “in range”.

#### 3.3.1. Effects on Extraction Yield

**Figure 3:**
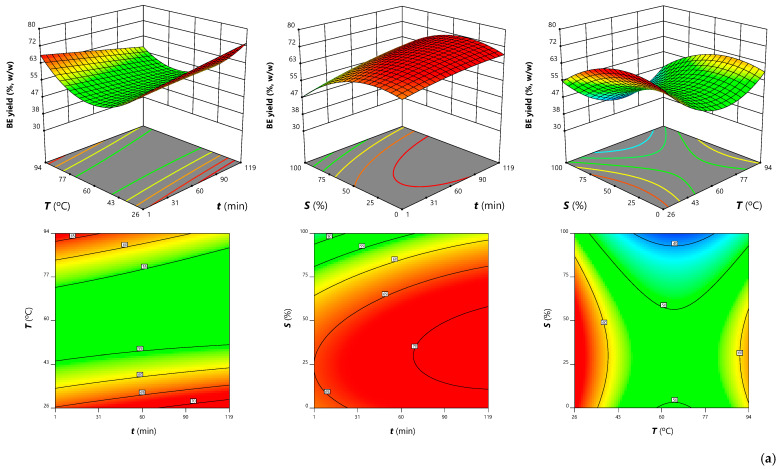

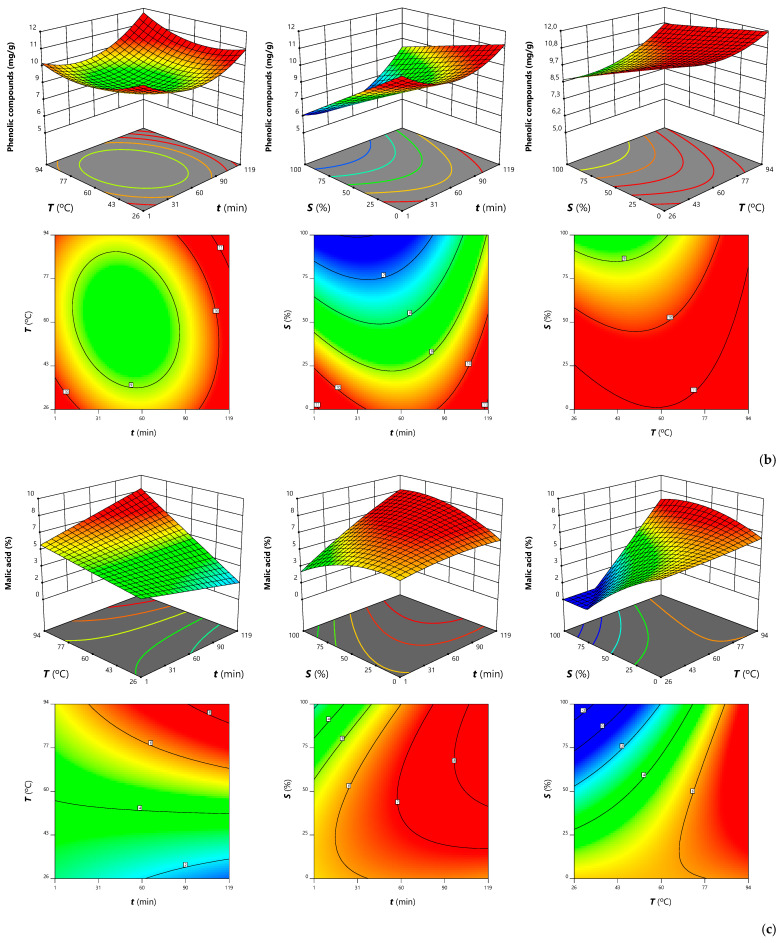
Response plots illustrating the effects of the independent variables on BE yield (**a**), phenolic compounds, (**b**) and malic acid (**c**) obtained from quince peel. In each plot, the unplotted independent variable was kept constant at its optimum shown in Table S5.

**Figure 4:**
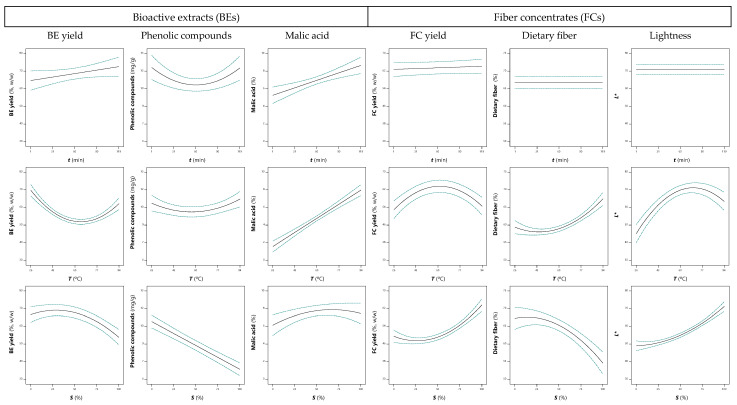
Response plots for the effects of each independent variable on extraction yields, phenolic compounds, and malic acid content of bioactive extracts (BEs), and dietary fiber content and lightness of fiber concentrates (FCs) obtained from quince peel. In each 2D plot, the unplotted independent variables were kept constant at their optimum shown in Table S5.

As shown in Table 3, the solvent was the variable that most affected the BE yield, through linear (3.81) and quadratic (−2.67) effects. Temperature ranked second with a linear effect (−1.98) and interacted with time (−1.54), a variable that was not significant (*p* < 0.05) but necessary for the model hierarchy. These extraction trends are visually represented in Figure 3 and Figure 4, where it can be seen that lower EtOH percentages and temperatures favored the BE yield, a result that can be due to the greater recovery of water-soluble compounds such as simple sugars. Furthermore, the time × temperature interaction (−1.54) seemed to promote BE yield with longer extraction times at low temperature or shorter times at higher temperature (Figure 3a). Thus, the optimum BE yield of 69 ±2% (*w/w*) was associated with quince peel processing at 26.7 °C for 64.4 min with 33.4% EtOH (Table S5).

#### 3.3.2. Effects on Phenolic Compounds

The phenolic compound groups quantified in quince peel BEs (Table 2) were fitted to Equation (1) to obtain the predictive models, whose coefficient are shown in Table 3. The solvent affected the recovery of phenolic compounds through linear effects (−1.03), as also verified for the three phenolic classes that constitute this group of bioactive compounds and for the BE yield. The quadratic effects of temperature and time were significant (*p* < 0.05), and this last variable interacted with the solvent. Thus, as shown in Figure 3 and Figure 4, more than one optimal point was found; while low EtOH percentages favored the extraction of phenolic compounds, both longer or shorter times and higher or lower temperatures seemed to lead to an increase in response values. Thus, the conditions that maximized the recovery of phenolic compounds to the maximum value of 10.6 ± 0.2 mg/g BE involved 64.2 min processing at 88.0 °C with 0% EtOH. Furthermore, conditions were also determined to maximize extraction as far as possible, while minimizing time and temperature. In this case, 9.6 ± 0.2 mg/g BE were associated with 8.5 min of extraction at 34.9 °C with 39.8% EtOH, conditions that reduce the time and temperature by approximately 87% and 60% with a loss of phenolic compounds of only about 9.4%.

As illustrated in Figure S2, flavan-3-ols and phenolic acids showed an extraction trend very similar to that described above for the total content of phenolic compounds. The main differences in the optimal extraction conditions of phenolic acids from quince peel were related to longer processing time (114 min) and those of flavan-3-ols and flavonols were differentiated by 0% EtOH (Table S5).

#### 3.3.3. Effects on Malic Acid and Other Organic Acids

The malic acid extraction patterns were characterized by the complexity of the model coefficients in Table 3, which showed interactions between all variables. The solvent induced linear (−1.44) and quadratic (−0.35) effects, but interacted positively with time (0.46) and temperature (0.86). As shown in Figure 3 by the response surface and projection areas in red, and also in Figure 4, intermediate percentages of EtOH appeared to favor malic acid extraction. In turn, higher concentrations were obtained when applying higher temperatures for longer times. Although the linear effect of time was not significant (*p* < 0.05), it interacted with the other two variables. Thus, short times worked better with lower EtOH percentages. For malic acid, the optimal processing conditions (87.7 min, 92.7 °C, 54.4% EtOH) yielded 7.9 ± 0.3 g/100 g BE (Table S5). In order to reduce the time and temperature involved in the extraction, a second optimization was performed to minimize its use. This objective was met using 10.0 min of extraction at 44.4 °C with 26.5% EtOH, yielding 6.1 ± 0.3 g/100 g of malic acid (about 23% less than under optimal conditions).

In addition to malic acid, the quinic acid and total organic acid contents in Table S3 were also fitted to Equation (1) to obtain the model coefficients shown in Table S4. While the extraction of total organic acids and malic acid has the same extraction behavior (given the high malic acid proportion), that of quinic acid differed mainly by the quadratic effect (0.10) of solvent and the absence of interactions between the variables (illustrated in Figure S3). In fact, as shown in Table S5, the optimal extraction conditions for quinic acid differ from those of malic acid and total organic acids mainly by the EtOH percentage.

#### 3.3.4. Effects on Soluble Sugars

Soluble sugars represented a considerable percentage of the quince peel BEs (Table S3). Therefore, when extracting bioactive compounds, it is important to know the qualitative and quantitative profile of sugars and how they are affected by processing. The effects of each independent variable on the extraction of soluble sugars are illustrated in Figure S3. As expected, these carbohydrates were best extracted using low EtOH percentages. The solvent interacted with the temperature, resulting in higher sugar levels when extracting at medium–high temperatures. The optimal processing conditions in Table S5 show that it is possible to obtain up to 65 ± 1 g of soluble sugars in 100 g of BE. However, it becomes more important to know the sugar content reached when applying the optimal extraction conditions defined for the target bioactive compounds. These may represent ~54–55% of the BE obtained under the condition defined for malic acid and phenolic compounds (Table S5), while the BE obtained for maximum yield can be composed of 60% of these molecules. The remaining fraction may correspond to other low molecular weight substances and pectin, which has about 78% of galacturonic acid in quince peel [45].

#### 3.3.5. Effects on Fiber Concentrates (FCs)

**Figure 5:**
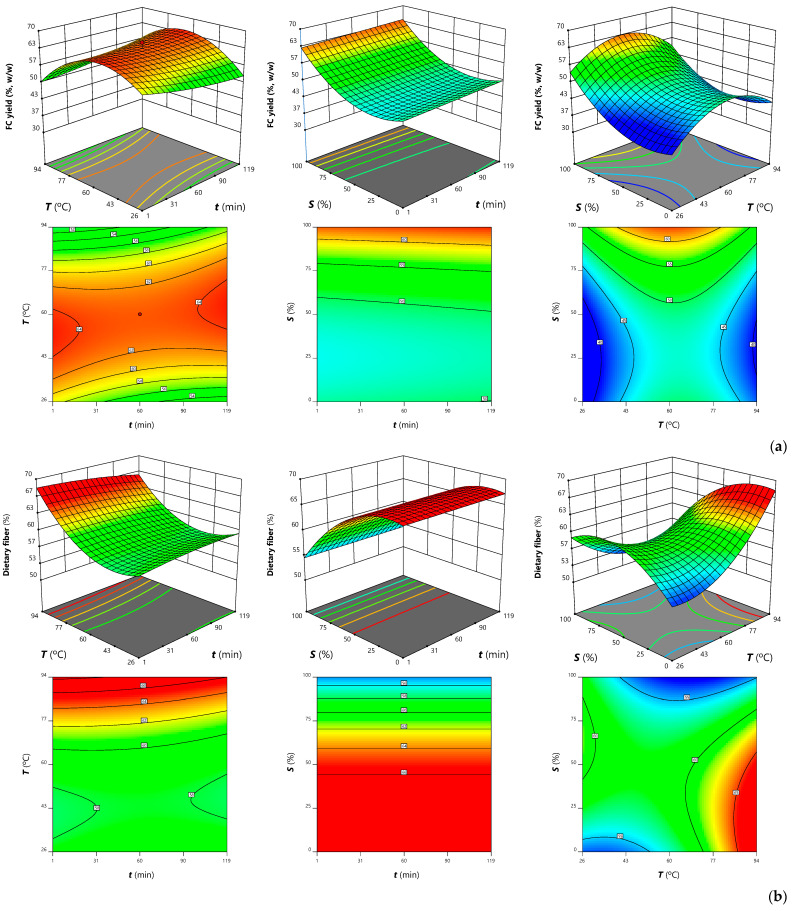
Response plots illustrating the effects of the independent variables on FC yield (**a**) and dietary fiber content (**b**) obtained from quince peel. In each plot, the unplotted independent variables was kept constant at its optimum, shown in Table S5.

In parallel with the production of BEs, FCs were obtained and characterized in terms of color and dietary fiber content. The parametric values translating the extraction trends are shown in Table 3. The most marked effects were induced by the solvent through positive linear (4.21) and quadratic (3.63) effects. Higher EtOH percentage were thus associated with higher FC yields, as illustrated in the response plots in Figure 5a (and in contrast to BE yield, as shown in Figure 4). It can also be noted that increasing the temperature to about 60 °C promoted the FC yield, which gradually decreased thereafter due to the quadratic effect. This variable interacted positively with time, which did not have a significant (*p* > 0.05) impact on extraction. The optimal extraction conditions in Table S5 (61.3 °C for 69.1 min with 99.9% EtOH) were associated with a maximum FC yield of 64 ± 2% (*w/w*).

When comparing the 3D plots in Figure 5 or the effects of each variable represented in Figure 4, it can be seen that the FC yield and dietary fiber do not follow the same trends. In general, the highest levels of dietary fiber were found in FCs obtained at high temperatures and medium–low EtOH percentages. In fact, only these two independent variables significantly impacted the extraction, through both linear and quadratic effects, but also interacted negatively (Table 3). Increasing temperature thus promoted dietary fiber content when using medium–low EtOH percentages, but was associated with lower response values when combined with high percentages (Figure 5). A reduction in dietary fiber with increasing EtOH percentage was also observed by Afifi et al. [43] in FCs resulting from date seed extractions. The FCs had 79.4% dietary fiber, resulting from a 3 h extraction with 25% EtOH at 55 °C; thus, although the FC yield was relatively low, it was of high purity. Li et al. [14] studied the supercritical water extraction of dietary fibers form date pits and reached higher yields when applying higher temperatures (~180 °C), while time (10–30 min) and feed mixture concentration (2–10%, *w/w*) did not induce significant variations. According to the authors, dietary fibers are thermo-stable within the tested range and degradation is not expected. In another work, Al-Farsi and Lee [13] quantified 57.9 g/100 g of total dietary fiber (52.7% insoluble fiber) in date seeds and 83.5 and 82.2 g/100 g of dietary fiber (about 81.5% insoluble fiber) in FCs obtained by water and 50% acetone extractions, respectively. This result was related to the extraction of phenolic compounds and other constituents, such as protein and soluble sugars, which lead to an increase in the insoluble dietary fiber (cellulose and hemicellulose). In turn, most of the soluble fiber (pectins, inulin, and gums) was recovered in BEs, which led to its reduction in the FCs.

The coefficients obtained for the FC lightness (L*) showed the significance of solvent and manly temperature through linear, quadratic, and integration terms (Table 3). As depicted in Figure 4, the trends recorded for FCs and L* were in agreement and, therefore, the lighter FCs had the highest yields. Contrariwise, the FCs with higher proportions of fiber were darker and obtained at > 80 °C with 20 or 50% EtOH. For redness (a*), the experimental data allowed the development of a predictive model counting only on three significant (*p* < 0.05) coefficients, among which the solvent × temperature interaction term (0.49) was the most expressive.

### 3.4. Experimental Validation of the Predictive Models

The optimal extraction conditions determined for phenolic compounds (9 min, 35 °C, 40% EtOH) and malic acid (88 min, 93 °C, 54% EtOH) (Table S5) were experimentally applied to obtain quince peel BEs with enriched contents of these bioactive constituents. For phenolic compounds, the optimal settings that minimized extraction time and temperature were selected. Furthermore, the condition for simultaneous extraction of phenolic compounds and malic acid was also determined (117 min, 79 °C, 45% EtOH) by simultaneously maximizing both response variables, keeping the standard error “in range”.

The post-analysis confirmation performed with Design-Expert software showed a good agreement between actual and model-predicted results at a 95% confidence level. As shown in Table 4, there were no significant differences between the BE and FC yields obtained with the three extractions. The phenolic BE contained about 13% more phenolic compounds than the other two BEs, given the higher flavan-3-ol and flavonol contents. On the other hand, the malic acid BE showed the highest levels of this compound and also phenolic acids, followed by the phenolic/malic acid BE. The settings defined for these two BEs seem to be suitable for the extraction of compounds with an organic carboxylic acid function, which was somehow expected as they do not differ much from those (114.3 min, 82.8 °C, 36.3% EtOH) of phenolic acids (Table S5).

The FCs also show a color within the expected range (Table 4); those resulting from the phenolic extraction were lighter (higher L* value) and yellower (higher b* value), while those from the malic acid extraction were the darkest. As discussed above, these color tones visually shown in Table 4 could probably be due to the different temperatures used in the extractions.

Overall, the suitability of the extraction processes developed to recover high value-added compounds from quince peel was demonstrated by these results. However, since it is difficult to obtain maximum yields with just one extraction setup, the one that targets the desired compound(s) or the one that is most sustainable for large-scale exploitation should be selected, taking into account the associated gains and losses.

### 3.5. Bioactive Properties of BEs Obtained under Optimized Conditions

#### 3.5.1. Antioxidant Activity

The quince peel BEs obtained under the optimized extraction conditions were evaluated for their antioxidant activity by testing their capacity to inhibit the TBARS formation and the oxidative hemolysis. As shown in Table 5, the best activity in both assays was shown by Trolox, a synthetic analog of alpha-tocopherol. In the TBARS assay, the malic acid BE best inhibited the formation of TBARS, followed by the phenolic/malic acid BE and then the phenolic BE. The antioxidant activity of the tested food additives was curiously lower than that of the quince peel BEs. E302 is a salt of ascorbic acid approved by EFSA as a food antioxidant [46], while E223 is a synthetic additive used as antioxidant, preservative, and disinfectant associated with some uncertainties about its reactivity in different foods and resulting reaction products [47]. Therefore, quince peel BEs may be more effective in delaying rancidity than the tested additives, thus representing a natural alternative with potential to be exploited by the food sector.

In a previous study, Othman et al. tested quince peel extracts obtained with boiling water and hydroethanolic extractions with the TBARS bioassay and obtained EC_50_ values of 39.2 and 60.3 µg/mL, respectively [9]. Therefore, better lipid peroxidation inhibition was achieved in the present work with the malic acid extract. In fact, malic acid and phenolic acids were very strongly (*R* = −0.964, *p* < 0.001) and strongly (*R* = −0.754, *p* < 0.05) correlated with this bioactivity, respectively (Table S6). Despite this, synergisms between antioxidant compounds may also occur.

Among the quince peel BEs, the malic acid BE was also the one that best protected RBCs from oxidative damage caused by AAPH-derived free radicals (Table 5), with an efficiency superior to that (160 µg/mL for a 60 min Δ*t* and 296 µg/mL for a 120 min Δ*t*) of the aqueous extract tested by Othman et al. [9], but comparable to that (115 µg/mL for a 60 min Δ*t* and 230 µg/mL for a 120 min Δ*t*) of its hydroethanolic extract. Then, came the phenolic and phenolic/malic acid BEs; although the phenolic BE was more efficient for the shorter Δ*t*, a similar protection (*p* > 0.05) for the 120 min Δ*t* was observed for both BEs. This antioxidant behavior was monitored with OxHLIA, since the protective action of antioxidant compounds depends on several factors, including short-term and long-term reaction kinetics. So, while some antioxidants can react faster and become depleted in the system, others can have a prolonged action over time. As shown in Table S6, malic acid correlated better than phenolic acids for longer exposure times.

The food additive E302 was more effective in protecting the RBC membrane than the quince peel BEs (Table 5). On the other hand, the E223 had no antihemolytic effects in the tested range of concentrations. According to previous reports, this sulfur-containing inorganic compound has the ability to induce the typical sickle shape of RBCs, thus compromising the integrity of their membrane and leading to their hemolysis [48,49], which may justify these results.

Regarding previous antioxidant activity studies, Magalhães et al. [50] correlated the antihemolytic activity of quince peel and pulp methanolic extracts with caffeoylquinic acids. Alesiani et al. [51] attributed strong free radical-scavenging activity to quercetin-type flavonols found in quince peel and a moderate capacity to other compounds, including phenolic acids. In general, greater antioxidant activity has been attributed to extracts from the peel than from the pulp of this fruit and related to the phenolic composition [11,52]. Despite this, Szychowski et al. [35] described how, although phenolic compounds contribute greatly to the antioxidant activity of the pulp, these phytochemicals are weakly correlated with the peel activity. Therefore, other compounds such as malic acid may be involved.

#### 3.5.2. Antimicrobial Activity

In addition to the antioxidant effects, it is also important that the natural ingredients to be used as food additives have antimicrobial properties to prevent microbial growth and food spoilage. The developed quince peel BEs and positive controls were tested against foodborne bacteria and micromycetes and the obtained results are shown in Table 6. Some extracts were more effective at inhibiting visible microbial growth and killing the original inoculum than at least one of the control food additives. For example, the three BEs were more effective than the sulfite-containing E224 against *B. cereus*, a Gram positive, spore-forming, and facultative anaerobic rod responsible for food poisoning, vomiting, and diarrhea [53]. E224 and malic acid BE had the same ability to inhibit the growth of *S. aureus*, the most dangerous staphylococcal bacteria, and *E. cloacae*. In turn, when compared to E211, these two bacteria were particularly well inhibited and killed by phenolic BE and mostly malic acid BE, as well as *E. cloacae* by malic acid BE. Furthermore, E211 had the same MIC and MBC values as the three BEs against *L. monocytogenes*, phenolic and malic acid BEs against *E. coli*, and malic acid BE against *S.* Typhimurium. Based on these observations, the antimicrobial activity of the quince peel BEs can be ranked as follows: malic acid BE > phenolic BE > phenolic/malic acid BE. It is also interesting to note that the MIC and MFC achieved with the malic acid BE were lower than those obtained previously by Othman et al. for the same bacterial strains [9].

The antifungal activity results of the quince peel BEs are also displayed in Table 6. The food additive E224, which offered greater antibacterial capacity, also stood out for its antifungal effect, although less effectively than the azole antifungals (except against *T. viride*). Both E224 and malic acid BE yielded the same MIC against the three *Aspergillus* spp. In turn, the MIC/MFC values reached for E211 were equal to those of malic acid e phenolic BEs for *A. fumigatus* and those of malic acid BE for *A. niger*, and superior to those of malic acid BE for *A. versicolor*. On the other hand, the BE dosage required to inhibit or kill *P. funiculosum* and *T. viride* was twice that of E211. Even so, the BEs were more active than ketoconazole against *T. viride*. Among the six micromycetes treated with quince peel BEs, *P. verrucosum* var. *cyclopium*, the fungus that produces the potent neurotoxin verrucosidin [54], appeared to have the greatest degree of resistance (MIC/MFC > 8 mg/mL).

The results of this work are comparable to those of Othman et al. [9], who reported slightly lower MIC and MFC for most of the tested fungal strains, except for *A. niger*, for which our extracts behaved better. Other studies have attributed greater antimicrobial activity to aqueous acetone extracts from quince peel than from its pulp, especially against *S. aureus* and *P. aeruginosa*, but also *E. coli* and *Candida albicans* [11]. Quince peel ethanolic extracts have also been described as having the ability to disrupt biofilms formed by *E. coli*, *L. monocytogenes*, *S. aureus*, and *S.* Typhimurium, with MIC ranging from 5 to 60 µg/mL [55]. According to other studies, although the entry of malic acid into the bacterial cell is limited by its low lipid solubility, it may enter the cells when undissociated and change the internal pH of the microorganism, causing antimicrobial effects [56]. These results suggested that quince peel BEs may be useful to prevent or fight some microbial contaminations. Even so, it will be important to elucidate which BE constituents contribute most to the antimicrobial effects and the involved mechanisms of action.

## 4. Conclusions

BEs and FCs were simultaneously obtained from quince peel through a “zero-waste” biorefinery approach. The BEs contained phenolic acids, flavan-3-ols, glycosylated flavonols, malic acid, fructose, and glucose, among other water-soluble constituents. The optimal extraction conditions for BE yield (66.4 min, 28.4 °C, and 42.6% EtOH), phenolic compounds (64.2 min, 88.0 °C, and 0% EtOH) and malic acid (87.7 min, 92.7 °C, and 54.4% EtOH) were associated with response values of 69% (*w/w*), 10.6 mg/g BE, and 7.9 g/100 g BE, respectively. For FCs, higher yields tended to be associated with lower fiber percentages, and those containing more dietary fiber were darker. The maximum fiber content (67 g/100 g FC) was associated with the use of 92.2 °C and 35.5% EtOH. The conditions foreseen for the extraction of phenolic compounds and malic acid were experimentally validated and the BEs obtained showed ability to inhibit lipid peroxidation and oxidative hemolysis and antimicrobial potential against foodborne bacteria and fungi. Furthermore, these BEs stood out in some aspects compared to synthetic food additives, especially the malic acid-enriched BE. Overall, the bioactive and functional food extracts resulting from the total upcycling of quince peel could be used as natural ingredients for food preservation and fortification. In future studies, it will be interesting to investigate the effect of solid/liquid ratio on extraction yields and assess the stability and preservative and fortifying capacity of the extracts in food matrices, including beverages.

## Figures and Tables

**Table 1:** Coded and decoded values of the independent variables combined in the CCRD.

Independent Variables	Symbols	Units	Levels ^1^				
			−1.68	−1	0	+1	+1.68
*X*_1_: Time	*t*	min	1	25	60	95	119
*X*_2_: Temperature	*T*	°C	26	40	60	80	94
*X*_3_: EtOH percentage	*S*	%, *v/v*	0	20	50	80	100

^1^ Each independent variable was studied at five coded levels (−1.68, −1, 0, +1, +1.68).

**Table 2:** Extraction yields, phenolic compounds and malic acid contents of bioactive extracts (BEs), and dietary fiber content and color parameters of fiber concentrates (FCs) obtained experimentally from quince peel with the 20 runs of the CCRD.

Run	Experimental Domain			Bioactive Extract (BE) Dependent Variables						Fiber Concentrate (FC) Dependent Variables					
	*t* (min)	*T* (°C)	*S* (%)	BE yield (%)	PC (mg/g)	PA (mg/g)	F3O (mg/g)	FL (mg/g)	MA (g/100 g)	FC yield (%)	DF (g/100 g)	L*	a*	b*	RGB Color
1	25	40	20	58.55	9.77	3.26	4.36	2.15	6.43	43.66	57.84	59.66	7.48	21.22	
2	95	40	20	59.12	9.99	3.72	4.17	2.10	4.59	43.08	56.78	56.75	7.50	21.14	
3	25	80	20	57.89	9.48	3.39	4.08	2.02	6.43	42.11	64.44	37.24	6.85	13.97	
4	95	80	20	53.24	10.13	3.74	4.45	1.94	6.63	46.76	62.65	35.62	5.44	11.59	
5	25	40	80	50.31	6.86	2.26	2.68	1.92	0.59	51.90	57.27	55.88	7.20	20.91	
6	95	40	80	54.12	7.95	2.76	3.38	1.81	0.79	51.59	58.66	56.02	7.62	21.57	
7	25	80	80	50.59	7.10	2.20	2.88	2.02	4.32	49.41	57.73	59.51	7.92	21.57	
8	95	80	80	47.30	8.89	3.08	3.85	1.96	6.18	52.70	57.53	58.60	8.14	19.93	
9	1	60	50	48.36	8.93	2.82	3.86	2.25	3.95	51.64	57.21	58.35	6.52	21.61	
10	119	60	50	48.62	10.04	3.71	4.23	2.10	5.07	50.25	58.64	56.80	6.31	20.41	
11	60	26	50	67.09	8.87	3.26	3.76	1.85	2.90	39.82	59.83	51.76	6.81	22.31	
12	60	94	50	59.19	9.04	3.39	3.83	1.82	6.97	40.81	65.55	34.09	8.51	20.18	
13	60	60	0	49.69	9.78	3.56	4.13	2.09	6.06	49.18	55.33	55.90	7.43	21.07	
14	60	60	100	34.47	6.50	2.00	2.62	1.88	1.63	65.53	52.46	70.62	6.63	22.25	
15	60	60	50	49.69	8.43	2.90	3.50	2.03	4.60	50.31	59.95	54.21	7.49	21.30	
16	60	60	50	51.82	7.67	2.61	3.08	1.98	4.72	48.18	58.11	56.62	7.71	21.39	
17	60	60	50	53.32	8.48	3.03	3.40	2.05	5.37	46.68	59.35	56.42	6.91	20.79	
18	60	60	50	51.73	7.71	2.64	3.07	2.00	4.57	48.27	57.86	57.30	7.22	21.29	
19	60	60	50	49.46	7.80	2.64	3.07	2.09	4.75	50.54	57.94	58.92	7.05	21.40	
20	60	60	50	52.03	8.17	2.88	3.34	1.94	5.41	47.97	60.48	58.33	7.20	21.54	

*t*: time; *T*: temperature; *S*: solvent (EtOH percentage); PC: phenolic compounds; PA: phenolic acids; F3O: flavan-3-ols; FL: flavonols; MA: malic acid; DF: dietary fiber; L*: chromaticity from ^(0)^black to ^(100)^white; a*: chromaticity from ^(−)^green to ^(+)^red; and b*: chromaticity from ^(−)^blue to ^(+)^yellow; RGB (red, green, blue) color was obtained from CIELAB color values.

**Table 3:** Parametric values estimated with Equation (1) and statistical data of the models’ fitting procedure for BEs and their bioactive constituents and for FCs and their dietary fiber content and color parameters.

Coefficients		BE Yield	PC	PA	F3O	FL	MA	FC Yield	DF	L*	a*
Intercept	*b*_0_	50.89 ± 0.65	8.05 ± 0.11	2.77 ± 0.05	3.28 ± 0.07	2.01 ± 0.01	4.83 ± 0.10	49.05 ± 0.59	58.79 ± 0.36	56.86 ± 0.57	7.23 ± 0.14
Linear terms	*b*_1 (*t*)_	−0.23 ± 0.51 *	0.41 ± 0.08	0.27 ± 0.04	0.18 ± 0.05	−0.04 ± 0.01	0.17 ± 0.09 *	0.35 ± 0.46 *	ns	ns	−0.08 ± 0.11 *
	*b*_2 (*T*)_	−1.93 ± 0.51	0.10 ± 0.08 *	0.05 ± 0.04 *	0.06 ± 0.05 *	−0.01 ± 0.01 *	1.32 ± 0.09	0.18 ± 0.46 *	1.57 ± 0.28	−4.91 ± 0.44	0.25 ± 0.11
	*b*_3 (*S*)_	3.81 ± 0.51	−1.03 ± 0.08	−0.47 ± 0.04	−0.50 ± 0.05	−0.06 ± 0.01	−1.44 ± 0.09	4.21 ± 0.46	−1.12 ± 0.28	4.80 ± 0.44	0.02 ± 0.11 *
Quadratic terms	*b*_11 (*tt*)_	ns	0.48 ± 0.08	0.15 ± 0.04	0.27 ± 0.05	0.06 ± 0.01	ns	ns	ns	ns	−0.24 ± 0.11
	*b*_22 (*TT*)_	ns	0.29 ± 0.08	0.17 ± 0.04	0.18 ± 0.05	−0.06 ± 0.01	ns	−3.40 ± 0.44	1.55 ± 0.27	−5.37 ± 0.43	ns
	*b*_33 (*SS*)_	−2.67 ± 0.49	ns	ns	ns	ns	−0.35 ± 0.09	3.63 ± 0.44	−1.56 ± 0.27	1.82 ± 0.43	ns
Interaction terms	*b*_12 (*tT*)_	−1.54 ± 0.67	ns	ns	ns	ns	0.46 ± 0.13	1.10 ± 0.59	ns	ns	ns
	*b*_13 (*tS*)_	ns	0.25 ± 0.11	ns	0.19 ± 0.07	0.07 ± 0.01	0.46 ± 0.13	ns	ns	ns	ns
	*b*_23 (*TS*)_	ns	ns	ns	ns	ns	0.89 ± 0.13	ns	−1.64 ± 0.37	6.22 ± 0.58	0.49 ± 0.14
**Modeling statistics**											
Model F-value		34.18	38.18	49.38	26.43	27.81	71.54	31.97	28.10	108.00	4.63
Model *p*-value		<0.0001	<0.0001	<0.0001	<0.0001	<0.0001	<0.0001	<0.0001	<0.0001	<0.0001	0.0099
Lack-of-Fit		0.2237	0.7923	0.8772	0.5805	0.9774	0.6356	0.3454	0.6731	0.5502	0.1723
*R* ^2^		0.9404	0.9463	0.9463	0.9242	0.9277	0.9766	0.9365	0.9094	0.9749	0.6913
Adj *R*^2^		0.9129	0.9215	0.9272	0.8893	0.8944	0.9629	0.9072	0.8770	0.9657	0.5342
Ad. Precision		27.29	20.83	25.04	17.17	19.14	28.23	24.41	23.56	40.83	7.77
C.V. (%)		3.64	3.59	4.65	5.24	1.84	7.77	3.48	1.76	3.01	5.56

In each term, parametric subscripts 1, 2, and 3 stand for the independent variables time (*t*), temperature (*T*), and solvent (*S*), respectively. PC: phenolic compounds; PA: phenolic acids; F3O: flavan-3-ols; FL: flavonols; MA: malic acid; DF: dietary fiber; *R*²: coefficient of determination; *R*²_ajd_: adjusted coefficient of determination; Ad. Precision: Adequate precision; C.V.: coefficient of variation (%). * Not significant but necessary for model hierarchy. L*: chromaticity from ^(0)^black to ^(100)^white; a*: chromaticity from ^(−)^green to ^(+)^red.

**Table 4:** Extraction yields, contents of phenolic compounds and malic acid in the bioactive extracts (BEs), and color of the fiber concentrates (FCs) obtained from quince peel under optimal extraction conditions defined for phenolic compounds, malic acid, and both constituents.

	Phenolic Extraction	Malic Acid Extraction	Phenolic/Malic Acid Extraction
BE yield (%, *w/w*)	48 ± 3 ^a^	49 ± 3 ^a^	50 ± 2 ^a^
Phenolic compounds (mg/g BE)	9.3 ± 0.6 ^a^	8.1± 0.4 ^b^	8.1 ± 0.6 ^b^
Phenolic acids (mg/g BE)	2.8 ± 0.3 ^b^	3.5 ± 0.2 ^a^	3.1 ± 0.3 ^a,b^
Flavan-3-ols (mg/g BE)	4.2 ± 0.4 ^a^	3.0 ± 0.2 ^b^	3.7 ± 0.3 ^b^
Flavonols (mg/g BE)	2.3 ± 0.3 ^a^	1.62 ± 0.01 ^b^	1.30 ± 0.03 ^c^
Malic acid (g/100 g BE)	4.5 ± 0.2 ^c^	7.6 ± 0.4 ^a^	6.0 ± 0.1 ^b^
FC yield (%, *w/w*)	51 ± 1 ^a^	50 ± 8 ^a^	47 ± 1 ^a^
L* value	54.9 ± 0.7 ^a^	35.8 ± 0.2 ^c^	41.4 ± 1.1 ^b^
a* value	7.0 ± 0.2 ^a^	7.5 ± 0.9 ^a^	8.2 ± 0.2 ^a^
b* value	23.3 ± 0.5 ^a^	17.1 ± 0.3 ^c^	19.4 ± 0.5 ^b^
RGB color			

L*: chromaticity from ^(0)^black to ^(100)^white; a*: chromaticity from ^(−)^green to ^(+)^red; b*: chromaticity from ^(−)^blue to ^(+)^yellow; RGB (red, green, blue) color. In each line, different letters indicate statistically significant differences (*p* < 0.05) between samples.

**Table 5:** Antioxidant activity of phenolic, malic acid, and phenolic/malic acid-enriched quince peel bioactive extracts (BEs) and positive controls (calcium ascorbate (E223), sodium metabisulfite (E302), and Trolox).

Bioassays		Bioactive Extracts (BEs)			Positive Controls		
		Phenolic BE	Malic Acid BE	Phenolic/Malic Acid BE	E302	E223	Trolox
TBARS (EC_50_, µg/mL)		83 ± 1 ^d^	26 ± 1 ^b^	45 ± 2 ^c^	284 ± 9 ^f^	229 ± 9 ^e^	5.4 ± 0.3 ^a^
OxHLIA	Δ*t* 60 min	187 ± 9 ^d^	105 ± 3 ^c^	223 ± 7 ^e^	41 ± 1 ^b^	na	21.7 ± 0.4 ^a^
(IC_50_ µg/mL)	Δ*t* 120 min	471 ± 12 ^d^	234 ± 5 ^c^	458 ± 9 ^d^	84 ± 2 ^b^	na	43 ± 1 ^a^

In each line, different letters indicate statistically significant differences (*p* < 0.05) between samples. na: no antihemolytic activity at the tested concentrations.

**Table 6:** Antibacterial and antifungal activity of phenolic, malic acid, and phenolic/malic acid-enriched quince peel bioactive extracts (BEs) and positive controls (sodium benzoate (E211), potassium metabisulfite (E224), streptomycin, ampicillin, ketoconazole, and bifonazole).

Microorganisms	Bioactive Extracts (BEs)						Positive Controls							
	Phenolic BE		Malic Acid BE		Phenolic/Malic Acid BE		E211		E224		Streptomycin		Ampicillin	
	MIC	MBC	MIC	MBC	MIC	MBC	MIC	MBC	MIC	MBC	MIC	MBC	MIC	MBC
Gram-positive bacteria														
*Staphylococcus aureus*	4	8	1	2	4	8	4	4	1	1	0.04	0.1	0.25	0.45
*Bacillus cereus*	1	2	1	2	1	2	0.5	0.5	2	4	0.1	0.2	0.25	0.4
*Listeria monocytogenes*	1	2	1	2	1	2	1	2	0.5	1	0.2	0.3	0.4	0.5
Gram-negative bacteria														
*Escherichia coli*	1	2	1	2	4	8	1	2	0.5	1	0.2	0.3	0.4	0.5
*Salmonella* Typhimurium	2	4	1	2	4	8	1	2	1	1	0.2	0.3	0.75	1.2
*Enterobacter cloacae*	1	2	0.5	1	4	8	2	4	0.5	0.5	0.2	0.3	0.25	0.5
											**Ketoconazole**		**Bifonazole**	
	MIC	MFC	MIC	MFC	MIC	MFC	MIC	MFC	MIC	MFC	MIC	MFC	MIC	MFC
*Aspergillus fumigatus*	1	2	1	2	2	4	1	2	1	1	0.25	0.5	0.15	0.2
*Aspergillus niger*	2	4	1	2	2	4	1	2	1	1	0.2	0.5	0.15	0.2
*Aspergillus versicolor*	>8	>8	1	2	4	8	2	2	1	1	0.2	0.5	0.1	0.2
*Penicillium funiculosum*	2	4	2	4	2	4	1	2	0.5	0.5	0.2	0.5	0.2	0.25
*Penicillium verrucosum* var. *cyclopium*	>8	>8	>8	>8	>8	>8	2	4	1	1	0.2	0.3	0.1	0.2
*Trichoderma viride*	2	4	2	4	2	4	1	2	0.5	0.5	2.5	3.5	0.2	0.25

The results are presented as minimum inhibitory (MIC) and minimum bactericidal (MBC) or fungicidal (MFC) concentrations (mg/mL); 30% EtOH used as a negative control did not have any influence on the microbial growth at the maximum concentration (40 μL of 30% EtOH per 100 μL).

## Data Availability

Not applicable.

## Supplementary Materials

The following supporting information can be downloaded at: https://www.mdpi.com/article/10.3390/antiox12020260/s1, Table S1: Phenolic compound identified in quince peel extracts; Table S2: Contents (mg/g BE) of individual phenolic compounds obtained experimentally from quince peel; Table S3: Contents (g/100 g BE) of quinic acid, total organic acids, fructose, glucose, sucrose, and total soluble sugars obtained experimentally from quince peel; Table S4: Parametric values estimated with the polynomial Equation (1) and statistical data of the models’ fitting procedure for sugars and acids; Table S5: Optimal conditions for extraction of quince peel constituents and respective response values; Table S6: Pearson's correlation coefficients (R) of extract constituents with antioxidant activity; Figure S1: Representative chromatogram of the phenolic profile of the quince peel extract obtained with the 19th run of the design matrix recorded at 280 nm (a) and 370 nm (b). Peak identification is shown in Table S1; Figure S2: Response surface plots illustrating the effects of the independent variables on the extraction of phenolic acids, flavan-3-ols, flavonols from quince peel. In each 3D plot, the excluded variable is fixed at its optimal value; Figure S3: Response plots for the effects of each independent variable on the extraction of organic acids and soluble sugars from quince peel. In each 2D plot, the excluded variables are fixed at their optimal value.
