# Supplementary material for: Upcycling Quince Peel into Bioactive Ingredients and Fiber Concentrates through Multicomponent Extraction Processes
Source: Antioxidants (Basel). 2023 Jan 23;12(2):260. doi: 10.3390/antiox12020260 (PMC9952593; doi:10.3390/antiox12020260)
Supplement: Supplementary file 1 [file antioxidants-12-00260-s001.zip › antioxidants-2101477-supplementary.pdf]

## Supplementary Material

**Table S1.** Phenolic compound identified in quince peel extracts.

| **Peak** | **R*t*** (min) | **$\lambda_{max}$** (nm) | **[M-H]$^-$** (*m/z*) | **Tentative identification** |
|---|---|---|---|---|
| 1 $^A$ | 4.43 | 324 | 353 | *cis*-3-*O*-Caffeoylquinic acid |
| 2 $^A$ | 4.39 | 325 | 353 | *trans*-3-*O*-Caffeoylquinic acid |
| 3 $^B$ | 5.85 | 292 | 337 | 3-*O*-*p*-Coumaroylquinic acid |
| 4 $^A$ | 6.56 | 326 | 353 | *cis*-5-*O*-Caffeoylquinic acid |
| 5$^A$ | 7.43 | 323 | 353 | *trans*-5-*O*-Caffeoylquinic acid |
| 6 $^C$ | 8.63 | 275 | 289 | (+)-Catechin |
| 7 $^*$ | 10.47 | 311 | 337 | 5-*O*-*p*-Coumaroylquinic acid |
| 8 $^C$ | 11.08 | 276 | 865 | β-Type (epi)catechin trimer |
| 9 $^C$ | 12.52 | 272 | 1153 | β-Type (epi)catechin tetramer |
| 10 $^*$ | 13.83 | 298 | 577 | β-Type (epi)catechin dimer |
| 11 $^C$ | 14.16 | 280 | 1153 | β-Type (epi)catechin tetramer |
| 12 $^*$ | 14.57 | 271 | 865 | β-Type (epi)catechin trimer |
| 13 $^C$ | 15.10 | 275 | 865 | β-Type (epi)catechin trimer |
| 14 $^*$ | 15.76 | 275 | 865 | β-Type (epi)catechin trimer |
| 15 $^D$ | 16.70 | 355 | 609 | Quercetin-*O*-deoxyhexosil-hexoside |
| 16 $^*$ | 17.52 | 268 | 863 | Procyanidin with A-type linkage |
| 17 $^D$ | 19.86 | 357 | 593 | Kaempferol-*O*-deoxyhexosil-hexoside |

R*t*: retention time; $\lambda_{max}$: wavelengths of maximum absorption in the UV-vis region; [M-H]$^-$: deprotonated ion. Standards used in quantification: $^A$ chlorogenic acid; $^B$ *p*-coumaric acid; $^C$ catechin; and $^D$ quercetin-3-*O*-glucoside. $^*$Not used in the optimization process.

**Table S2.** Contents (mg/g BE) of individual phenolic compounds obtained experimentally from quince peel.

| Runs | Phenolic compounds | | | | | | | | | | | |
|---|---|---|---|---|---|---|---|---|---|---|---|---|
| | **1** | **2** | **3** | **4** | **5** | **6** | **8** | **9** | **11** | **13** | **15** | **17** |
| **1** | 0.530 | 0.733 | 0.172 | 1.425 | 0.400 | 0.653 | 1.542 | 0.931 | 0.589 | 0.645 | 1.233 | 0.917 |
| **2** | 0.597 | 0.721 | 0.213 | 1.751 | 0.435 | 0.693 | 1.471 | 0.909 | 0.490 | 0.608 | 1.292 | 0.812 |
| **3** | 0.549 | 0.730 | 0.195 | 1.517 | 0.397 | 0.653 | 1.459 | 0.862 | 0.538 | 0.563 | 1.188 | 0.828 |
| **4** | 0.544 | 0.825 | 0.184 | 1.756 | 0.432 | 0.744 | 1.559 | 0.947 | 0.541 | 0.658 | 1.262 | 0.679 |
| **5** | 0.394 | 0.483 | 0.112 | 0.989 | 0.279 | 0.421 | 0.884 | 0.562 | 0.365 | 0.446 | 1.148 | 0.777 |
| **6** | 0.433 | 0.587 | 0.148 | 1.259 | 0.332 | 0.576 | 1.165 | 0.718 | 0.385 | 0.536 | 1.031 | 0.779 |
| **7** | 0.490 | 0.524 | 0.094 | 0.745 | 0.347 | 0.446 | 0.935 | 0.638 | 0.409 | 0.451 | 1.049 | 0.971 |
| **8** | 0.552 | 0.658 | 0.173 | 1.349 | 0.349 | 0.555 | 1.332 | 0.849 | 0.504 | 0.615 | 1.193 | 0.767 |
| **9** | 0.385 | 0.585 | 0.150 | 1.424 | 0.276 | 0.565 | 1.402 | 0.772 | 0.501 | 0.621 | 1.490 | 0.760 |
| **10** | 0.616 | 0.752 | 0.222 | 1.701 | 0.422 | 0.646 | 1.446 | 0.890 | 0.617 | 0.627 | 1.339 | 0.762 |
| **11** | 0.504 | 0.644 | 0.182 | 1.591 | 0.339 | 0.612 | 1.236 | 0.821 | 0.471 | 0.624 | 1.204 | 0.646 |
| **12** | 0.501 | 0.718 | 0.186 | 1.677 | 0.304 | 0.554 | 1.292 | 0.893 | 0.484 | 0.605 | 1.158 | 0.666 |
| **13** | 0.580 | 0.915 | 0.191 | 1.481 | 0.397 | 0.594 | 1.378 | 0.879 | 0.583 | 0.701 | 1.213 | 0.873 |
| **14** | 0.343 | 0.521 | 0.107 | 0.774 | 0.254 | 0.412 | 0.888 | 0.530 | 0.380 | 0.411 | 1.137 | 0.740 |
| **15** | 0.475 | 0.732 | 0.125 | 1.251 | 0.316 | 0.548 | 1.269 | 0.739 | 0.436 | 0.511 | 1.240 | 0.785 |
| **16** | 0.599 | 0.642 | 0.097 | 0.880 | 0.395 | 0.436 | 1.059 | 0.667 | 0.445 | 0.473 | 1.045 | 0.935 |
| **17** | 0.550 | 0.724 | 0.102 | 1.329 | 0.325 | 0.512 | 1.324 | 0.696 | 0.420 | 0.450 | 1.206 | 0.847 |
| **18** | 0.550 | 0.634 | 0.099 | 0.937 | 0.420 | 0.445 | 1.025 | 0.659 | 0.432 | 0.509 | 1.070 | 0.933 |
| **19** | 0.366 | 0.644 | 0.122 | 1.192 | 0.315 | 0.406 | 1.176 | 0.684 | 0.369 | 0.434 | 1.336 | 0.754 |
| **20** | 0.436 | 0.652 | 0.122 | 1.353 | 0.321 | 0.486 | 1.280 | 0.706 | 0.390 | 0.483 | 1.164 | 0.781 |

Compound **1**: *cis*-3-*O*-caffeoylquinic acid; compound **2**: *trans*-3-*O*-caffeoylquinic acid; compound **3**: 3-*O*-*p*-coumaroylquinic acid; compound **4**: *cis*-5-*O*-caffeoylquinic acid; compound **5**: *trans*-5-*O*-caffeoylquinic acid; compound **6**: (+)-catechin; compound **8**: β-type (epi)catechin trimer; compound **9**: β-type (epi)catechin tetramer; compound **11**: β-type (epi)catechin tetramer; compound **13**: β-type (epi)catechin trimer; compound **15**: quercetin-*O*-deoxyhexosil-hexoside; compound **17**: kaempferol-*O*-deoxyhexosil-hexoside.

**Table S3.** Contents (g/100 g BE) of quinic acid, total organic acids, fructose, glucose, sucrose, and total soluble sugars obtained experimentally from quince peel.

| Run | Experimental domain | | | Bioactive extract (BE) dependent variables | | | | | |
|---|---|---|---|---|---|---|---|---|---|
| | *t* (min) | *T* (°C) | *S* (%) | Quinic acid | Total organic acids | Fructose | Glucose | Sucrose | Total soluble sugars |
| 1 | 25 | 40 | 20 | 0.277 | 6.71 | 42.19 | 13.45 | 5.30 | 60.94 |
| 2 | 95 | 40 | 20 | 0.200 | 4.79 | 40.98 | 12.03 | 5.85 | 58.86 |
| 3 | 25 | 80 | 20 | 0.480 | 6.91 | 41.91 | 13.46 | 7.41 | 62.78 |
| 4 | 95 | 80 | 20 | 0.410 | 7.04 | 39.32 | 11.81 | 6.30 | 57.43 |
| 5 | 25 | 40 | 80 | 0.000 | 0.59 | 39.43 | 10.96 | 6.45 | 56.84 |
| 6 | 95 | 40 | 80 | 0.000 | 0.79 | 36.95 | 9.89 | 6.36 | 53.20 |
| 7 | 25 | 80 | 80 | 0.270 | 4.59 | 36.79 | 9.94 | 5.78 | 52.51 |
| 8 | 95 | 80 | 80 | 0.392 | 6.57 | 32.92 | 7.69 | 3.73 | 44.34 |
| 9 | 1 | 60 | 50 | 0.240 | 4.19 | 38.23 | 12.81 | 6.37 | 57.41 |
| 10 | 119 | 60 | 50 | 0.404 | 5.47 | 36.30 | 10.14 | 5.50 | 51.94 |
| 11 | 60 | 26 | 50 | 0.000 | 2.90 | 42.17 | 12.01 | 5.60 | 59.78 |
| 12 | 60 | 94 | 50 | 0.413 | 7.38 | 40.35 | 10.99 | 5.77 | 57.11 |
| 13 | 60 | 60 | 0 | 0.620 | 6.68 | 41.84 | 11.39 | 5.92 | 59.15 |
| 14 | 60 | 60 | 100 | 0.200 | 1.83 | 33.99 | 6.12 | 5.13 | 45.24 |
| 15 | 60 | 60 | 50 | 0.139 | 4.74 | 39.69 | 10.91 | 5.38 | 55.98 |
| 16 | 60 | 60 | 50 | 0.000 | 4.72 | 37.80 | 9.88 | 4.72 | 52.40 |
| 17 | 60 | 60 | 50 | 0.260 | 5.63 | 37.30 | 10.61 | 4.31 | 52.22 |
| 18 | 60 | 60 | 50 | 0.070 | 4.64 | 36.79 | 9.24 | 4.53 | 50.56 |
| 19 | 60 | 60 | 50 | 0.000 | 4.75 | 38.79 | 11.16 | 4.40 | 54.35 |
| 20 | 60 | 60 | 50 | 0.000 | 5.41 | 39.08 | 10.70 | 5.54 | 55.32 |

*t*: time; *T*: temperature; *S*: solvent (EtOH percentage).

**Table S4.** Parametric values estimated with the polynomial Equation (1) and statistical data of the models' fitting procedure for sugars and organic acids.

| Coefficients | | Quinic acid | Total organic acids | Fructose | Glucose | Sucrose | Total soluble sugars |
|---|---|---|---|---|---|---|---|
| Intercept | $b_0$ | 0.11 ± 0.03 | 4.99 ± 0.10 | 37.89 ± 0.27 | 10.40 ± 0.24 | 4.81 ± 0.17 | 53.64 |
| Linear terms | $b_1$ | 0.02 ± 0.02* | 0.19 ± 0.09* | -0.98 ± 0.26 | -0.80 ± 0.16 | -0.30 ± 0.11 | -2.08 ± 0.48 |
| | $b_2$ | 0.13 ± 0.02 | 1.45 ± 0.09 | -0.85 ± 0.26 | -0.38 ± 0.16 | -0.03 ± 0.11* | -1.26 ± 0.48 |
| | $b_3$ | -0.10 ± 0.02 | -1.54 ± 0.09 | -2.31 ± 0.26 | -1.55 ± 0.16 | -0.28 ± 0.11 | -4.14 ± 0.48 |
| Quadratic terms | $b_{11}$ | 0.07 ± 0.02 | ns | ns | 0.49 ± 0.15 | 0.42 ± 0.11 | ns |
| | $b_{22}$ | ns | ns | 1.10 ± 0.25 | 0.50 ± 0.15 | 0.34 ± 0.11 | 1.87 ± 0.63 |
| | $b_{33}$ | 0.10 ± 0.02 | -0.25 ± 0.09 | ns | -0.47 ± 0.15 | 0.28 ± 0.11 | ns |
| Interaction terms | $b_{12}$ | ns | 0.48 ± 0.13 | ns | ns | -0.45 ± 0.14 | ns |
| | $b_{13}$ | ns | 0.50 ± 0.13 | ns | ns | ns | ns |
| | $b_{23}$ | ns | 0.93 ± 0.13 | -0.59 ± 0.33 | ns | -0.73 ± 0.14 | -1.70 ± 0.63 |
| **Modeling statistics** | | | | | | | |
| Model F-value | | 13.41 | 78.28 | 26.02 | 23.56 | 9.51 | 24.81 |
| Model *p*-value | | <0.0001 | <0.0001 | <0.0001 | <0.0001 | 0.0006 | <0.0001 |
| Lack-of-Fit | | 0.7304 | 0.7700 | 0.7938 | 0.8672 | 0.9139 | 0.7804 |
| $R^2$ | | 0.8273 | 0.9786 | 0.9028 | 0.9322 | 0.8737 | 0.8986 |
| Adj $R^2$ | | 0.7656 | 0.9661 | 0.8681 | 0.8923 | 0.7819 | 0.8624 |
| Adequate precision | | 13.26 | 29.44 | 16.47 | 18.53 | 12.89 | 16.32 |
| C.V. (%) | | 42.25 | 7.58 | 2.45 | 5.39 | 7.43 | 3.23 |

In each term, parametric subscripts 1, 2, and 3 stand for the independent variables time (*t*), temperature (*T*), and solvent (*S*), respectively. $R^2$: coefficient of determination; $R^2_{ajd}$: adjusted coefficient of determination; C.V.: coefficient of variation (%). *Not significant but necessary for model hierarchy.

**Table S5.** Optimal conditions for extraction of quince peel constituents and respective response values.

| Responses | Optimal processing conditions | | | Response optimum |
|---|---|---|---|---|
| | $X_1$: ***t*** (min) | $X_2$: ***T*** (°C) | $X_3$: ***S*** (%, *v*/*v*) | Model-predicted values |
| BE yield | 66.4 | 26.7 | 33.4 | 69 ± 2% (*w*/*w*) |
| Phenolic compounds | 64.2 | 88.0 | 0.0 | 10.6 ± 0.2 mg/g |
| Phenolic compounds # | 8.5 | 34.9 | 39.5 | 9.6 ± 0.2 mg/g |
| Phenolic acids | 114.3 | 82.8 | 36.3 | 4.1 ± 0.1 mg/g |
| Flavan-3-ols | 40.4 | 85.9 | 0.0 | 4.7 ± 0.2 mg/g |
| Flavonols | 10.6 | 52.8 | 0.0 | 2.31 ± 0.03 mg/g |
| Malic acid | 87.7 | 92.7 | 54.4 | 7.9 ± 0.3 g/100 g |
| Malic acid # | 10.0 | 44.4 | 26.5 | 6.1 ± 0.3 g/100 g |
| Quinic acid | 96.7 | 91.8 | 8.5 | 0.74 ± 0.07 g/100 g |
| Organic acids | 89.2 | 92.5 | 56.2 | 8.2 ± 0.3 g/100 g |
| Fructose | 1.0 | 69.2 | 0.0 | 43.7 ± 0.7 g/100 g |
| Glucose | 7.4 | 36.5 | 34.1 | 14.3 ± 0.5 g/100 g |
| Sucrose | 18.4 | 76.7 | 16.8 | 7.8 ± 0.3 g/100 g |
| Soluble sugars | 5.01 | 70.7 | 0.0 | 65 ± 1 g/100 g |
| FC yield | 69.1 | 61.3 | 99.9 | 64 ± 2% (*w*/*w*) |
| Dietary fiber | - | 92.2 | 35.5 | 67 ± 1 g/100 g |
| Lightness (*L**) | - | 65.9 | 99.2 | 71 ± 1 (0–100) |

*t*: time; *T*: temperature; *S*: solvent (EtOH percentage). The standard error was kept "in range" in all cases.
# Extraction conditions obtained by minimizing the time and temperature values.
- The variable was not significant ($p < 0.05$).

**Table S6.** Pearson's correlation coefficients (*R*) of extract constituents with antioxidant activity.

| | | TBARS | OxHLIA | |
|---|---|---|---|---|
| | | | **Δ*t* 60** | **Δ*t* 120** |
| Phenolics compounds | *R* | 0.654 | -0.207 | 0.0117 |
| | Sig. | 0.056 | 0.593 | 0.763 |
| Phenolics acids | *R* | **-0.754*** | **-0.566** | **-0.720*** |
| | Sig. | 0.019 | 0.112 | 0.029 |
| Flavan-3-ols | *R* | 0.864** | 0.103 | 0.427 |
| | Sig. | 0.003 | 0.792 | 0.252 |
| Flavonols | *R* | 0.777* | -0.109 | 0.236 |
| | Sig. | 0.014 | 0.780 | 0.542 |
| Malic acid | *R* | **-0.964**** | **-0.660** | **-0.875**** |
| | Sig. | 0.000 | 0.053 | 0.002 |

Very strong correlation: ≥ 0.9 (■); Strong correlation: 0.7–0.9 (■); Moderate correlation: 0.5–0.7 (■).
*Significant at $p \leq 0.05$. **Significant at $p \leq 0.01$.

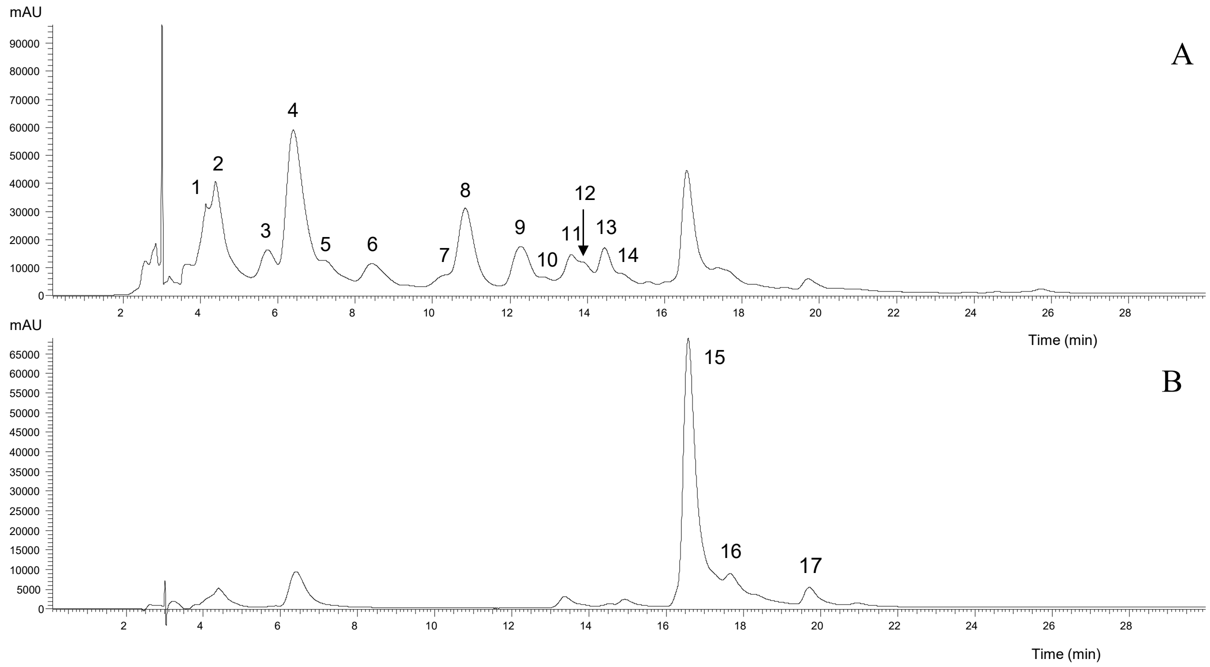


**Figure S1:** Representative chromatogram of the phenolic profile of the quince peel extract obtained with the 19th run of the design matrix recorded at 280 nm (a) and 370 nm (b). Peak identification is shown in Table S1.

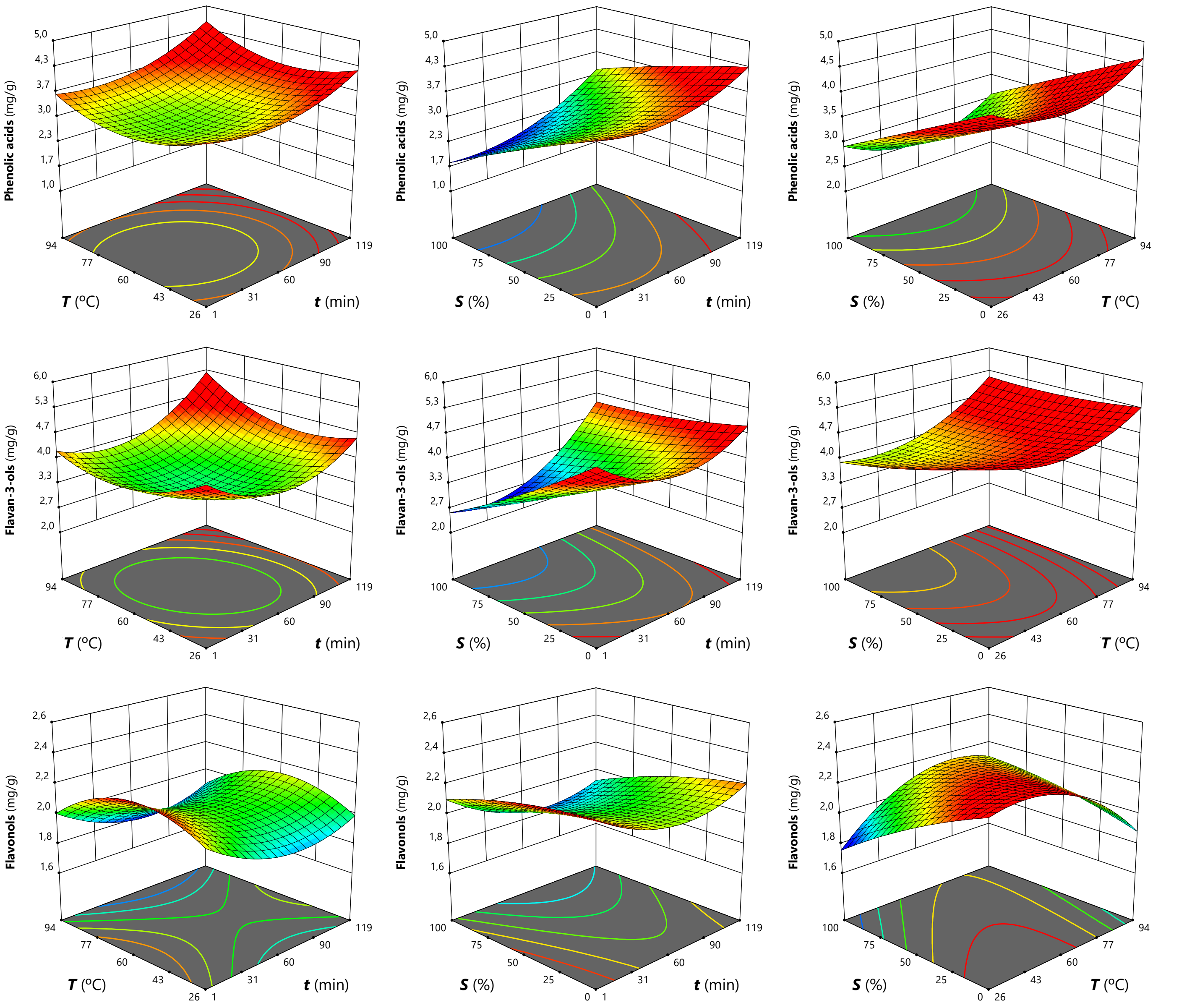


**Figure S2.** Response surface plots illustrating the effects of the independent variables on the extraction of phenolic acids, flavan-3-ols, flavonols from quince peel. In each 3D plot, the unplotted independent variable was kept constant at its optimal value in Table S4.

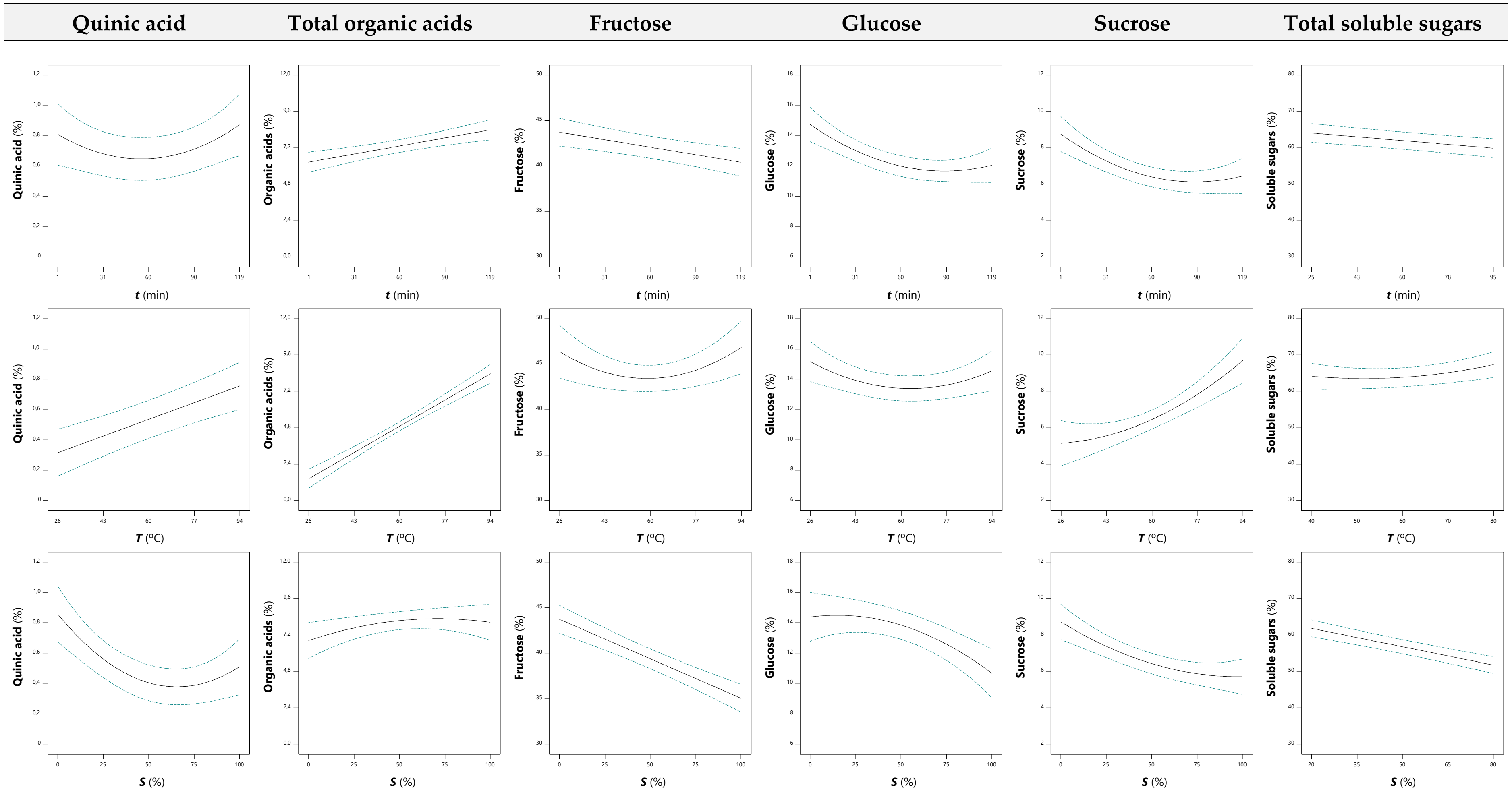


**Figure S3.** Response plots for the effects of each independent variable on the extraction of organic acids and soluble sugars from quince peel. In each 2D plot, the unplotted independent variable were kept constant at their optimal value in Table S4.
